# Fungal Diversity Associated with Thirty-Eight Lichen Species Revealed a New Genus of Endolichenic Fungi, *Intumescentia* gen. nov. (Teratosphaeriaceae)

**DOI:** 10.3390/jof9040423

**Published:** 2023-03-29

**Authors:** Hongli Si, Yichen Wang, Yanyu Liu, Shiguo Li, Tanay Bose, Runlei Chang

**Affiliations:** 1 College of Life Sciences, Shandong Normal University, Jinan 250014, China; 2 Department of Biochemistry, Genetics & Microbiology, Forestry and Agricultural Biotechnology Institute (FABI), University of Pretoria, Pretoria 0002, South Africa

**Keywords:** Ascomycota, China, lichens, multi-gene phylogeny, Mycosphaerellales

## Abstract

Fungi from the Teratosphaeriaceae (Mycosphaerellales; Dothideomycetes; Ascomycota) have a wide range of lifestyles. Among these are a few species that are endolichenic fungi. However, the known diversity of endolichenic fungi from Teratosphaeriaceae is far less understood compared to other lineages of Ascomycota. We conducted five surveys from 2020 to 2021 in Yunnan Province of China, to explore the biodiversity of endolichenic fungi. During these surveys, we collected multiple samples of 38 lichen species. We recovered a total of 205 fungal isolates representing 127 species from the medullary tissues of these lichens. Most of these isolates were from Ascomycota (118 species), and the remaining were from Basidiomycota (8 species) and Mucoromycota (1 species). These endolichenic fungi represented a wide variety of guilds, including saprophytes, plant pathogens, human pathogens, as well as entomopathogenic, endolichenic, and symbiotic fungi. Morphological and molecular data indicated that 16 of the 206 fungal isolates belonged to the family Teratosphaeriaceae. Among these were six isolates that had a low sequence similarity with any of the previously described species of Teratosphaeriaceae. For these six isolates, we amplified additional gene regions and conducted phylogenetic analyses. In both single gene and multi-gene phylogenetic analyses using ITS, LSU, SSU, RPB2, TEF1, ACT, and CAL data, these six isolates emerged as a monophyletic lineage within the family Teratosphaeriaceae and sister to a clade that included fungi from the genera *Acidiella* and *Xenopenidiella*. The analyses also indicated that these six isolates represented four species. Therefore, we established a new genus, *Intumescentia* gen. nov., to describe these species as *Intumescentia ceratina*e, *I. tinctorum*, *I. pseudolivetorum*, and *I. vitii*. These four species are the first endolichenic fungi representing Teratosphaeriaceae from China.

## 1. Introduction

Teratosphaeriaceae (Mycosphaerellales; Dothideomycetes; Ascomycota) [1] currently includes 61 genera and nearly 400 species [2,3]. This family encompasses fungi with a variety of lifestyles, such as saprophytes, extremophiles, human opportunistic, and plant pathogens [4]. Hence, these fungi are ubiquitous in distribution and have been reported from various extreme and atypical environments, such as leaf surfaces [5,6], sub-aerial biofilms [7], human teeth [8], arctic landscapes [9], tropical oligotrophic peatlands [10], and pyramids [11]. Concurrently, various fungi from Teratosphaeriaceae have also been recovered from lichens [12,13].

Lichen is a symbiotic relationship between fungi and algae or cyanobacteria, resulting in the formation of thalli [14,15,16]. The fungal diversity associated with lichens includes symbiotic, lichenicolous and endolichenic fungi [16]. Endolichenic fungi secrete a variety of secondary metabolites, some of which are beneficial to the survival of lichen in severe environments [17,18]. Endolichenic and lichenicolous fungi include species of the phyla Ascomycota, Basidiomycota, and Mucoromycota [16,17,19].

Globally, studies involving endolichenic fungi led to the identification and discovery of various new and previously identified fungal taxa representing various lineages of Ascomycota, such as Pezizomycetes, Dothideomycetes, Eurotiomycetes, Leotiomycetes, and Sordariomycetes, among others [20,21,22,23]. Echoing the global trend, several novel endolichenic fungal taxa were also identified in China [24,25,26,27,28]. In a recent fungal biodiversity study by Xu et al. (2022), the authors detected a substantial diversity of endolichenic fungi in the family Teratosphaeriaceae from *Heterodermia obscurata,* and proposed that Teratosphaeriaceae is likely to be one of the core endophytic fungal families associated with this lichen [13]. The authors used a high-throughput sequencing platform; hence, no isolates could be retrieved.

In this study, we aimed to catalogue the fungal diversity associated with lichen species from Yunnan Province of China. We selected this province as our sampling site because this region is considered a biodiversity hotspot due to its unique climatic conditions that support a diverse vegetation and, consequently, the microbes that are associated with it [29]. We hypothesized that the diversity of fungi that are associated with each lichen species will differ; however, there will be some overlap. This fungal diversity will include endolichenic fungi as well as other fungi, representing a wide range of ecological guilds. Therefore, we aimed to detect the diversity of fungal guilds within lichens. 

## 2. Materials and Methods

### 2.1. Collection and Identification of Lichen Samples

**Figure 1:**
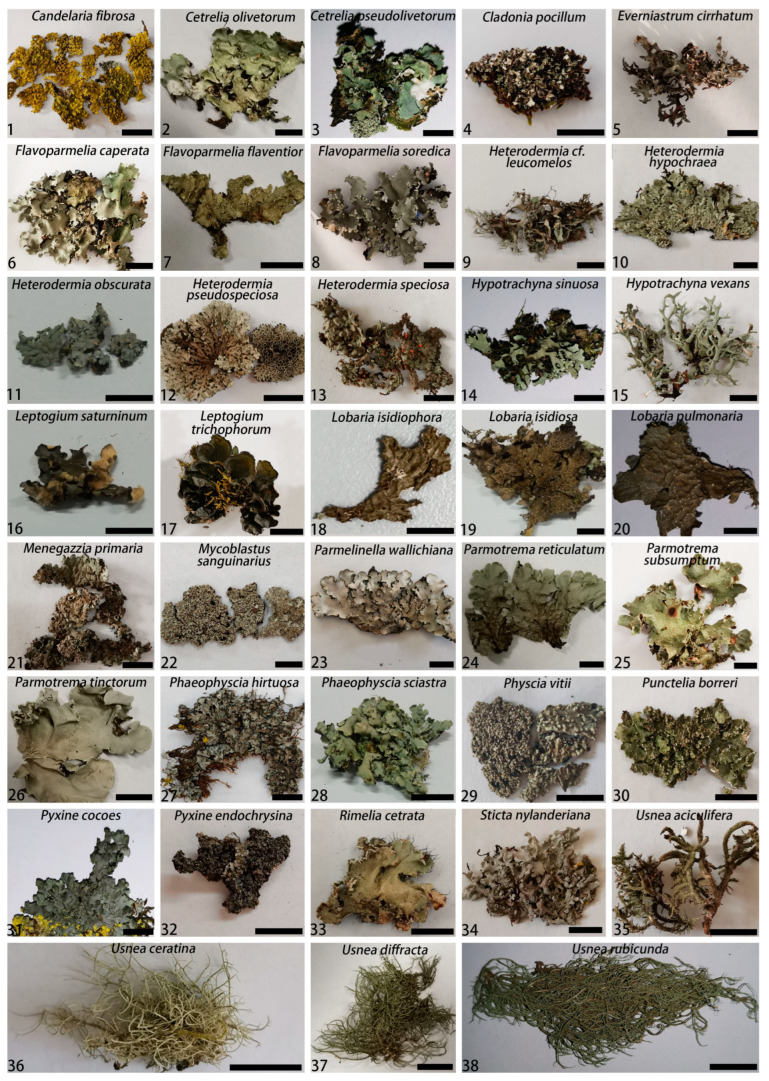
Morphology of 38 lichen samples collected in Yunnan Province in China between 2020 and 2021. During the species identification process, photographs were taken using the dried thallus samples. Bars = 1 cm.

**Figure 2:**
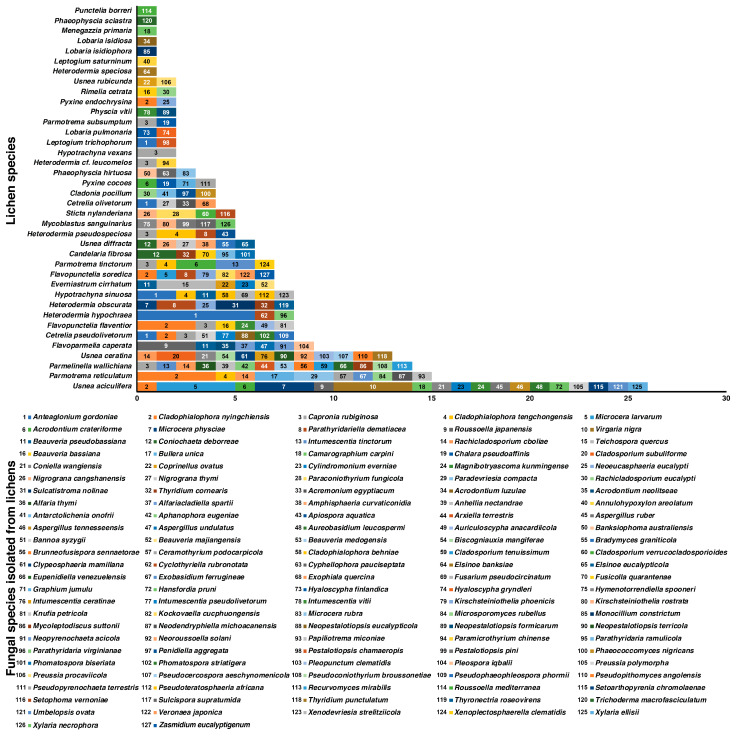
Fungi isolated from 38 lichen species sampled in this study from Yunnan Province, China, between 2020 and 2021. Fungal species are listed according to their abundance in lichens. Three most frequently isolated fungal species were *Anteaglonium gordoniae* (11 isolates), *Cladophialophora nyingchiensis* (11 isolates), and *Capronia rubiginosa* (nine isolates). Highest number of fungal isolates were recovered from the lichens *Usnea aciculifera* (26 isolates), *Parmotrema reticulatum* (15 isolates), *Parmelinella wallichiana* (14 isolates), and *Usnea ceratina* (13 isolates). The numbers within the bar-plot represent the fungal species.

Five surveys were conducted in Yunnan Province of China from 2020 to 2021. During these surveys, we collected 115 lichen samples representing different growth forms, such as crustose, foliose, and fruticose (Figure 1 and Figure 2, Table S1). We collected at least two thalli for each lichen. One of them was dried in a cabinet at 35 °C. This dried sample was intended for lichen photography and identification. The morphological identification of the lichen samples were carried out using the monographs published by Wang (2012) and Wang and Qian (2012) [30,31]. Following taxonomic identification of the lichens, all dried samples were discarded. A fresh lichen thallus was used for fungal isolation.

### 2.2. Isolation of Fungi from Lichen Thalli

All of the lichen samples collected during the surveys were asymptomatic. Hence, fungal isolation was conducted randomly from various parts of a thallus. The lichen samples were individually rinsed under running tap water and then with deionized sterile distilled water. Multiple 2 × 2 cm^2^ pieces of each lichen thallus were dissected under a Leica Zoom 2000 stereomicroscope. The upper and lower cortices of the thallus tissue were scraped off using a sterile blade and a pair of tweezers. The medullary layer was carefully removed and rinsed repeatedly with sterile deionized water. These medulla tissues from each lichen sample were then placed onto the surface of 2% potato dextrose agar medium (PDA, Qingdao Hope Bio-Technology Co., Ltd., Qingdao, China) amended with 0.05% streptomycin. All of the Petri plates were incubated at 25 °C in darkness. Hyphal tips of mycelia emerging from the medullary tissues were sub-cultured onto fresh PDA plates.

### 2.3. DNA Extraction, PCR Amplification, Sequencing, and Preliminary Identification

Total genomic DNA was extracted from 15-day old fungal cultures grown on PDA using a modified CTAB protocol [32]. For the purpose of the initial screening of all isolates, the complete internal transcribed spacer (ITS) and the partial nuclear large subunit ribosomal DNA (LSU) regions were amplified using primers ITS1/ITS4 [33] and LR0R/LR5 [33,34], respectively. 

For all novel fungal species, additional gene regions, such as RNA polymerase II (RPB2), translation elongation factor-1 α (TEF1), actin (ACT), and calmodulin (CAL) were amplified using the primers frpb2-5f/rpb2-7cr [35], 728F/2218R [36,37], ACT-512f/ACT2rd [36,37], and CAL-235F/CAL2RD [36,38], respectively. 

For all gene regions, each 25 μL of PCR reaction included 9.5 μL of water, 12.5 μL of 1-5^TM^ 2 × High-Fidelity Master Mix (buffer, MgCl_2_, dNTPs and Taq; Tsingke Co., China), 1 μL each of forward and reverse primers, and 1 μL DNA template. For all gene regions, PCR amplifications were conducted with an initial denaturation at 94 °C for 5 min, followed by 35 cycles of 94 °C for 30 s, 56 °C for 1 min, 72 °C for 90 s, and a final extension at 72 °C for 10 min. Positive amplification of the gene regions was determined using agarose gel electrophoresis. Samples were stained using Spark GoldView (SparkJade, China) and visualized under UV light.

All PCR products were sequenced by the Qingdao Sangong Biotechnology Co., LTD. The resulting forward and reverse sequences were assembled using Geneious v.10.2.2 (Biomatters, Auckland, New Zealand). Preliminary identification of the fungal isolates was carried out using the BLAST algorithm [39] available through the NCBI GenBank. Sequences from possibly novel fungus species were included in the phylogenetic analyses and were also submitted to GenBank (Table S2).

### 2.4. Guilds of Fungal Species Isolated from Lichens

Ecological guilds of the fungal species isolated from 38 lichen species were determined using the FungalTraits database [40]. Due to a paucity of data, the specific guild could not be identified for some fungal species. In those cases, we inserted an inquiry mark alongside the guild.

### 2.5. Phylogenetic Analyses

During preliminary identification of the fungal isolates using BLAST, six isolates emerged as potentially new fungal species from the family Teratosphaeriaceae. For the phylogenetic identification of these six isolates, separate datasets were prepared for all six gene regions: ITS (59 taxa), LSU (82 taxa), RPB2 (57 taxa), TEF1 (37 taxa), ACT (26 taxa), and CAL (29 taxa) (Table S2). The datasets include sequences generated in this study, as well as those from the ex-type isolates retrieved from GenBank and from a previous study by Wanasinghe et al. (2018) [3]. Irrespective of the datasets, *Staninwardia suttonii* served as an outgroup. All sequence datasets were aligned using MAFFT v.7 [41] and if needed, they were manually adjusted using MEGA v.7 [42]. 

All single genes and concatenated datasets were analyzed using the maximum likelihood (ML), Bayesian inference (BI), and maximum parsimony (MP) approaches. ML and BI were analyzed using the CIPRES Scientific Gateway platform [43]. Irrespective of the phylogenetic approaches, the appropriate nucleotide substitution model was determined using jModelTest v.2.1.6 [44]. The ML analysis was performed using RAxML v.8.2.12 with 1000 bootstrap replicates using GTR + Gamma as the substitution model [45]. For the BI analyses, MrBayes v.3.2.7 [46] ran 5 million generations from a random start tree with four MCMC chains using the substitution model GTR+I+G for ITS, GTR+G for LSU, TPM3uf+I+G for TEF, TIM3+I+G for ACT and RPB2, TIM1+I+G for CAL, and GTR+I+G for the concatenated dataset. For all analyses, the stop value was set to 0.01 and a temperature was set to 0.2, and the trees were sampled after every 100 generations. A quarter of the sampled trees were discarded during burn-in. The remaining trees were used for constructing consensus trees. MP analysis was performed using MEGA v.10.2.0 with 1000 bootstrapping replicates and gap as the fifth character state. The resulting trees from the ML, MP, and BI analyses were viewed using FigTree v.1.4 [47]. The alignments and trees were deposited in TreeBASE (Study ID29830).

### 2.6. Morphology and Growth Studies

Multiple approaches were used to induce sporulation in the novel fungal isolates. All isolates were sub-cultured on four microbial culture media. These were PDA, malt extract agar medium (MEA, Qingdao Hope Bio-technology, Qingdao, China), oat agar medium (OA, oats 20 g; agar, 20 g from Qingdao Hope Bio-technology, Qingdao, China; distilled water 1000 mL), and synthetic nutrient-poor agar medium (SNA; KH_2_PO_4_ 1 g; KNO_3_ 1 g; MgSO_4_7H_2_O 0.5 g; KCl 0.5 g; glucose 0.2 g from Qingdao Hope Bio-technology, Qingdao, China; sucrose 0.2 g; agar, 20 g from Qingdao Hope Bio-technology, Qingdao, China; distilled water 1000 mL) [48]. If any of the isolates did not sporulate on the above-mentioned media, then they were sub-cultured onto PDA, MEA, OA, and SNA amended with sterilized pine needles [49,50] and dried lichen powder (0.2 g/100 mL).

The micro-morphology of the potential new species was photographed using a Leica DM6 compound microscope attached to a Zeiss Axio Imager Z2 camera. Image J [51] was used to measure at least 50 readings for each taxonomically important attribute from all isolates.

For the growth study, all isolates from four potentially new species were sub-cultured onto PDA. All of the Petri plates were incubated at 25 °C for 15 days. Then, agar blocks measuring 5 mm in diameter were placed in the center of a 90 mm Petri dish. The isolates were incubated in three replicates at five different temperatures: 5, 10, 15, 20, 25, 30, and 35 °C (±0.5 °C). Colony diameter of each isolate was measured every two days until day 30.

Ex-holotype cultures of undescribed species were deposited at the Chinese General Library of Microbial Cultures (CGMCC), Beijing, China. Holotypic specimens were preserved in the culture collection of the Institute of Microbiology (HMAS), Beijing, China.

## 3. Results

### 3.1. Collection and Identification of the Lichen Samples

One hundred and fifteen lichens were classified into 38 species based on morphological identification using monographs. The most commonly collected lichen in this study was *Usnea aciculifera*, which is followed by *Flavoparmelia caperata* and *Usnea ceratina* (Table S1).

### 3.2. Isolation of the Fungi from Lichen Thalli and Preliminary Identification

In this study, we recovered a total of 205 fungal isolates representing 127 fungal species from phyla Ascomycota (118 species), Basidiomycota (eight species), and Mucoromycota (one species) (Figure 2, Table S3). Among these, three most frequently isolated fungal species were *Anteaglonium gordoniae* (Anteagloniaceae, Pleosporales; 11 isolates), *Cladophialophora nyingchiensis* (Herpotrichiellaceae, Chaetothyriales; 11 isolates), and *Capronia rubiginosa* (Herpotrichiellaceae, Chaetothyriales; nine isolates) (Figure 2, Table S3). The fungal diversity associated with lichens included both unique and overlapping taxa (Figure 2, Table S3). *Capronia rubiginosa*, *Cladophialophora nyingchiensis*, and *Anteaglonium gordoniae*, were isolated from eight, six, and five lichen species, respectively. Among the 38 lichen species sampled in this study, the highest number of fungal isolates were recovered from *Usnea aciculifera* (26 isolates), *Parmotrema reticulatum* (15 isolates), *Parmelinella wallichiana* (14 isolates), and *Usnea ceratina* (13 isolates) (Figure 2, Table S3).

Fungi isolated from these 38 lichen species represented a wide variety of guilds, such as saprophytes, plant pathogens, human pathogens, as well as entomopathogenic, endolichenic, and symbiotic fungi (Table S3). The majority of these fungi were saprophytes (80) followed by plant pathogens (24), entomopathogens (5), human pathogens (5), endolichenic fungi (4), endophytes (3), symbiotic fungi (3), parasites (2), fungicolous fungi (1), and insect-associated fungi (1) (Table S3).

During the preliminary identification, we detected 16 isolates from the Teratosphaeriaceae family. Among these were six isolates that had a low sequence similarity to previously described fungal species from this family. These were CGMCC3.23630 (*Usnea ceratina*), CGMCC3.23741 (*Physcia vitii*), CGMCC3.23636 (*Parmelinella wallichiana*), CGMCC3.23633 and CGMCC3.23634 (*Parmotrema tinctorum*), and CGMCC3.23635 (*Cetrelia pseudolivetorum*). 

### 3.3. Phylogenetic Analyses

**Figure 3:**
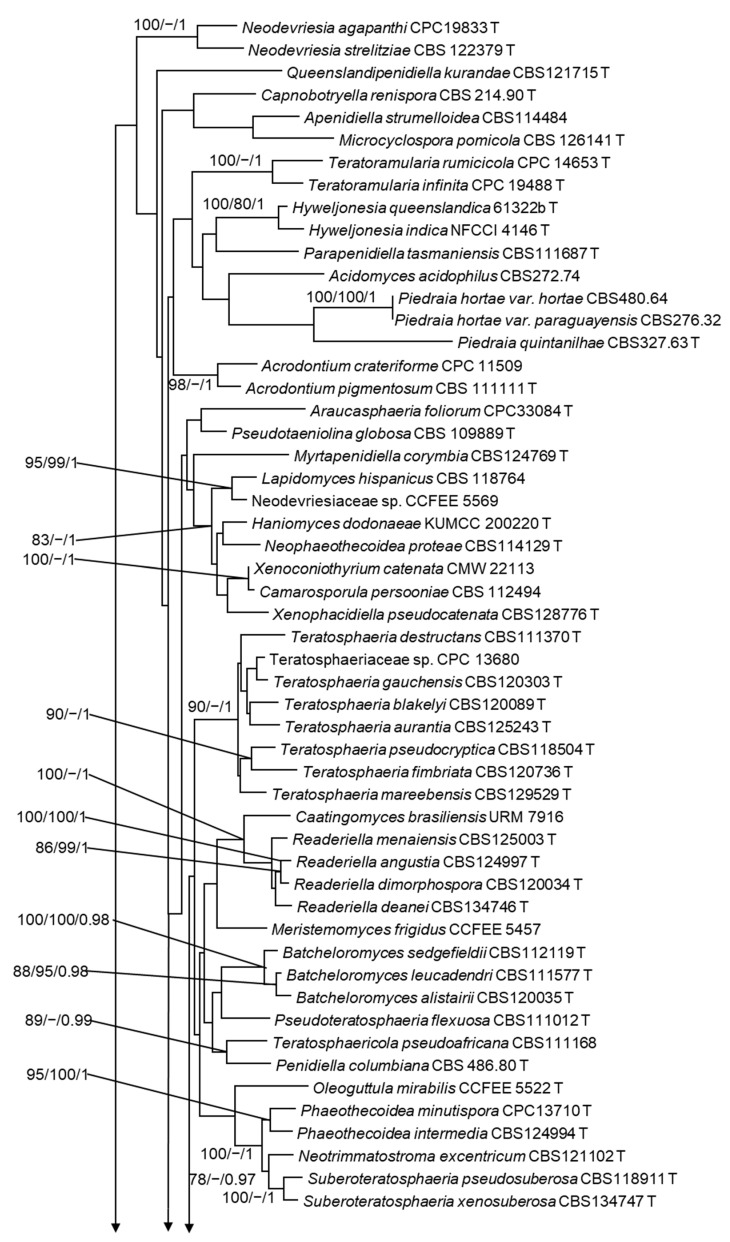

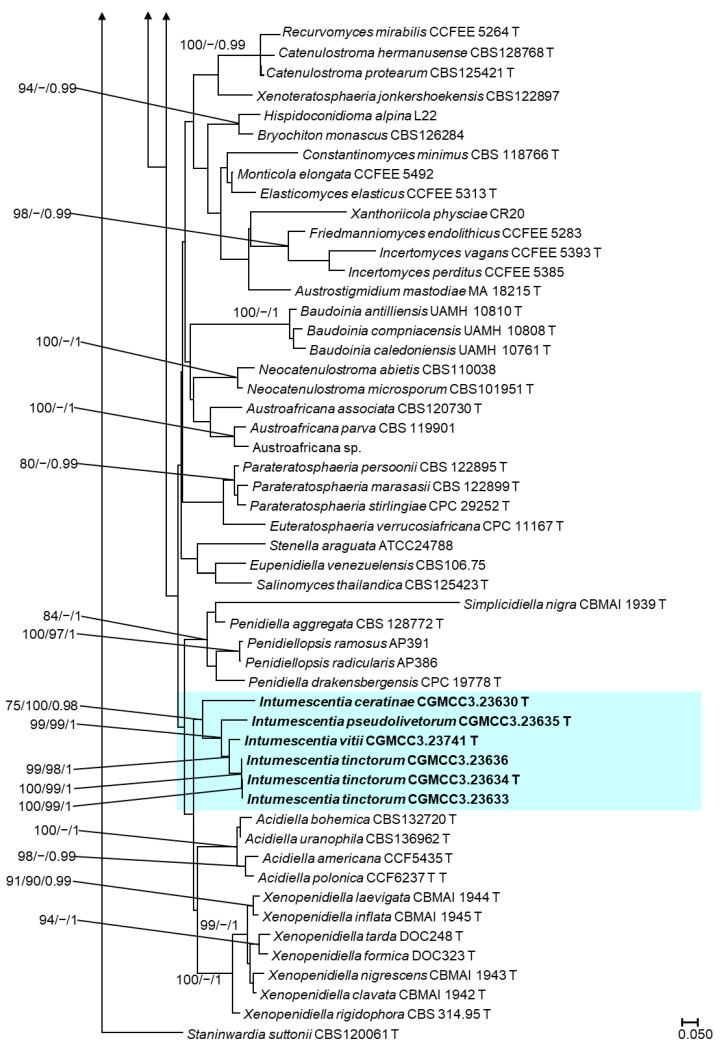
Maximum likelihood phylogeny using the concatenated dataset (LSU+ITS+ACT+ CAL+RPB2+TEF1) for Teratosphaeriaceae. The bootstrap support value ≥75% and posterior probability ≥ 0.95 displayed above the node are ML/MP/PP. The isolates of *Intumescentia* gen. nov. obtained in this study are shown in bold and highlighted in blue. T = ex-type isolates.

The concatenated dataset includes 116 taxa and 3541 characters including gaps (LSU: 1-882; ITS: 883-1539; ACT: 1540-2141; CAL: 2142-2559; RPB2: 2560-2991; TEF1: 2992-3541). In the ML analysis of the concatenated dataset, isolates CGMCC3.23630, CGMCC3.23741, CGMCC3.23636, CGMCC3.23633, CGMCC3.23634, and CGMCC3.23635 formed a monophyletic clade within the Teratosphaeriaceae family with significant branch support values (ML/MP/PP; 75/100/0.98; Figure 3). This clade of six isolates emerged as the sister to a clade that encompasses fungi from the genera *Acidiella* and *Xenopenidiella*, but this relationship did not receive significant branch support (Figure 3). In the phylogenetic analyses using single gene datasets, these six isolates formed a monophyletic clade in the ITS, LSU, ACT, and TEF trees (Figures S1–S6). However, this relationship was only significant in the TEF tree (Figure S3).

Within the clade that included the six previously undescribed species, CGMCC3.23633, CGMCC3.23634, and CGMCC3.23636 formed a monophyletic clade that received significant statistical support (ML/MP/PP; 100/99/1; Figure 3). The sequences for all amplified gene regions were identical between CGMCC3.23633 and CGMCC3.23634. However, when compared to CGMCC3.23636, it had two nucleotide differences in ITS and RPB2 and a single nucleotide difference in ACT, whereas there were no differences in LSU, CAL, and TEF1.

Isolate CGMCC3.23741 appeared as the sister to the clade that included CGMCC3.23633, CGMCC3.23634, and CGMCC3.23636. This relationship received high branch support (ML/MP/PP; 99/98/1; Figure 3). In addition to this, there were substantial differences in all gene regions between the two groups. With significant statistical support (ML/MP/PP; 99/99/1) and differences in the amplified gene regions, CGMCC3.23635 emerged as the sister to this clade (Figure 3). CGMCC3.23630 emerged as the basal diverging taxa within this clade (Figure 3). The isolate CGMCC3.23630′s phylogenetic position was a wobble in the single-gene trees. On the TEF tree, this isolate appeared as the sister taxon of a clade that included three *Baudoinia* species, but had no substantial branch support. Similarly, CGMCC3.23630 is either clustered with *Austroafricana associata* or appeared as an orphan in the CAL and RPB2 trees, respectively.

### 3.4. Taxonomy

***Intumescentia* **H. L. Si, R. L. Chang, T. Bose and Y. C. Wang, gen. nov. 

MycoBank No: 844851

Etymology: The name refers to the typical hyphal swellings that occur in this group of fungi. 

Description: The slow growing colonies on PDA are black-brown in color (top and reverse), compact, superficial, with usually gray or greenish black, tomentose, the margins may be entire or finely serrated and irregularly lobed. Hyphae are asperulous or smooth, brown in color, septate, multi-guttulate, and branched; their compartments are variable in size, usually with hyphal swelling that are apical or intercalary in position, with lateral branching usually arising from the swollen compartments. Conidial cells catenulate, three to eight or more in a chain, they are intercalary or apical in position, and are caducous. The conidia are columnar to doliiform in shape, dark brown in color, and basal and intercalary conidia have flat apices, and apical conidia with an acute apex are multi-guttulate. No sexual structures were observed.

Type species: *Intumescentia tinctorum* H. L. Si, R. L. Chang, T. Bose and Y. C. Wang

Notes: The genus *Intumescentia* displays a significant morphological variance with closely related genera *Acidiella*, *Araucasphaeria* and *Xenopenidiella*. In comparison to *Intumescentia*, *Acidiella,* and *Xenopenidiella,* they yield morphologically distinct mitospores [4,52]. *Acidiella* produces puffed and truncated arthroconidia, while *Xenopenidiella* produces branched chains of verruculose conidia that are ellipsoid to cylindrical-oblong in shape and brown in color. *Araucasphaeria* is known to produce sexual spores [53]. Fungi from the genera *Acidiella*, *Araucasphaeria*, and *Xenopenidiella* have a faster growth rate than *Intumescentia* [49,52,53]. 

**Figure 4:**
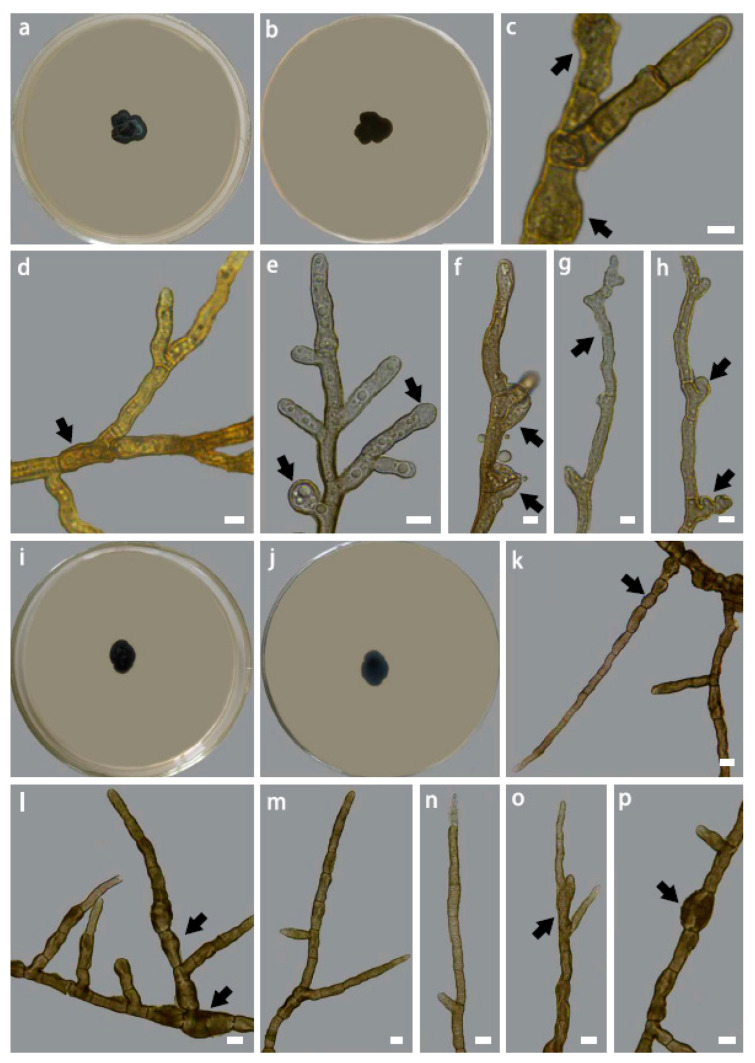
Colony morphology of *Intumescentia tinctorum* sp. nov. (CGMCC3.23634) on PDA medium after 30 days at 25 °C, (**a**) top, (**b**) reverse, hyphal morphology (**c**–**e**) branched hyphae with intercalary swelling, (**e**) apical hyphal swelling, (**f**) lateral branch emerging from swollen and distorted hyphal swelling, (**g**) slightly curved juvenile hyphae with distorted apex; (**h**) distorted lateral branching; colony morphology of *Intumescentia vitii* sp. nov. (CGMCC3.23741) on PDA medium after 30 days at 25 °C, (**i**) top, (**j**) reverse, hyphal morphology (**k**) peanut-shaped compartments, (**l**–**p**) a variety of swollen and distorted compartments with branches emerging from the base of septa. Bars = 5μm. Morphological structures listed above are indicated with arrows.

***Intumescentia tinctorum*** H. L. Si, R. L. Chang, T. Bose and Y. C. Wang, sp. nov. (Figure 4a–h)

MycoBank No: 844857

Holotype: China, Yunnan Province: Tiesuo Township, 26°32′71”N, 100°57′3”E, ca. 2115 m elev., isolated from *Parmotrema tinctorum*, 17 January 2021, H.L. Si, CX97C16 (HMAS 352171), ex-type Culture CGMCC3.23634. GenBank: ITS: OP345149, LSU: OP345116, SSU: OP345114, ACT: OP354478, CAL: OP354484, RPB2: OP354472, TEF1 OP354466.

Etymology: Named after its host lichen species, *Parmotrema tinctorum*.

Description: Hyphae are smooth, light brown in color, septate, multi-guttulate, and branched; compartments are variable in size and are often distorted, measuring 2.01–5.05 μm ($\bar{x}$ = 3.23 μm, n = 50), usually with hyphal swelling that is globose to sub-globose in shape, and apical or intercalary in position, and lateral branching usually arises from swollen or distorted compartments; juvenile hyphae are usually slightly curved. Asexual and sexual structures were not observed.

Colony morphology and growth: Colony on PDA after 30 days at 20 °C is black-brown in color (top and reverse), compact, with a surface that is greenish gray in color, minutely tomentose, margins are entire and irregularly lobed. Optimal growth temperature is 20 °C (0.31 mm/day). Growth was observed at 5 °C (0.06 mm/day), whereas no growth was detected at 35 °C.

Additional specimens examined: China, Yunnan Province: Tiesuo Township, 26°32′71” N, 100°57′3” E, ca. 2115 m elev., isolated from *Parmotrema tinctorum*, 17 January 2021, H.L. Si, CX97C1, ex-type Culture CGMCC3.23633, GenBank: ITS: OP345115, LSU: OP345111, SSU: OP345113, ACT: OP354477, CAL: OP354483, RPB2: OP354471, TEF1: OP354465; China, Yunnan Province: Tiesuo Township, 26°32′71” N, 100°57′3” E, ca. 2115 m elev., isolated from *Parmotrema wallichiana*, 17 January 2021, H.L. Si, CX96C5, ex-type Culture CGMCC3.23636, GenBank: ITS: OP289531, LSU: OP326178, SSU: OP326174, ACT: OP354476, CAL: OP354482, RPB2: OP354470, TEF1: OP354464.

Notes: In the phylogenetic analyses of single genes and concatenated datasets, the three isolates of *I. tinctorum* emerged as a monophyletic lineage (Figure 3). This fungal species shares both congruent and distinct morphological characteristics with closely related species (Table 1). Additionally, *I. tinctorum* has the lowest optimal growth temperature compared to other species in this genus (Table 1).

***Intumescentia vitii*** H. L. Si, R. L. Chang, T. Bose and Y. C. Wang, sp. nov. (Figure 4i–p)

MycoBank No: 844854

Holotype: China, Yunnan Province: Tiesuo Township, 26°32′71” N, 100°57′3” E, ca.2115 m elev., isolated from *Physcia vitii*, 17 January 2021, H.L. Si, CX89C2 (HMAS 352148, Holotype), ex-type Culture CGMCC3.23741, GenBank: ITS: OP342841, LSU: OP345120, SSU: OP345110, ACT: OP354481, CAL: OP354487, RPB2: OP354475, TEF1: OP354469. 

Etymology: Named after its host lichen species, *Physcia vitii*.

Description: Hyphae are smooth, dark brown in color, branched, septate, with constricted septa; the compartment is often peanut-shaped, multi-guttulate with guttles small in size; the compartment is variable in size and often distorted, measuring 2.64–7.31 μm ($\bar{x}$ = 4.09 μm, n = 50), usually with irregular globose hyphal swellings that are intercalary in position, with lateral branching usually arising below the septa. Juvenile hyphae and hyphal apices are thin walled and hyaline to light brown in color; matured hyphae have thick walls and are dark brown in color. Asexual and sexual structures were not observed.

Colony morphology and growth: Colony on PDA after 30 days at 25 °C is blackish brown in color (top and reverse), compact, superficial, tomentose, and margins are entire and irregularly lobed. Optimal growth temperature is 25 °C (0.18 mm/day). Growth was observed at 5 °C (0.06 mm/day), whereas no growth was detected at 35 °C.

Notes: *Intumescentia vitii* appeared as the sister of *I. tinctorum* in the ML (Figure 3), MP, and BI trees. These two species exhibit characteristics that are both overlapping and distinctive (Table 1). In addition to the DNA data, these two species can be differentiated through colony morphologies, hyphal dimensions, and optimal growth temperatures.

**Figure 5:**
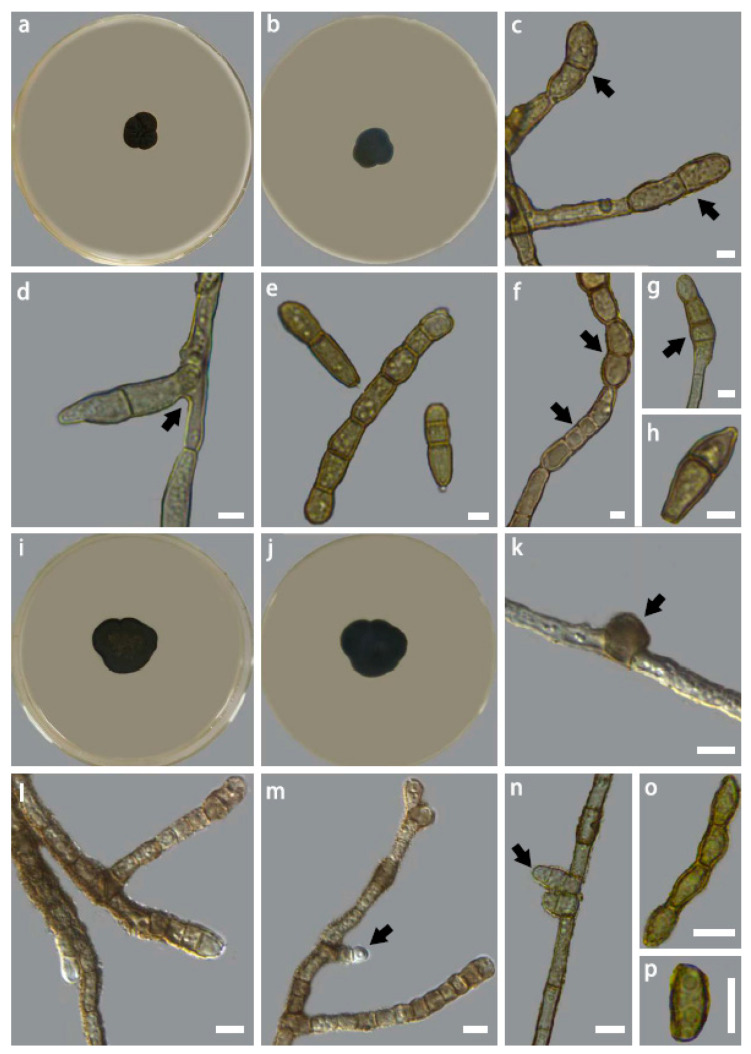
Colony morphology of *Intumescentia pseudolivetorum* sp. nov. (CGMCC3.23635) on PDA medium after 30 days at 25 °C, (**a**) top, (**b**) reverse, (**c**) hyphae with thick-walled apical conidia, (**d**) hyphae with lateral branching with basal constriction, (**e**) 2, 3, and 7-celled conidia, (**f**) chain of conidia with both columnar and fusoid conidia, (**g**) apical conidia, (**h**) a single fusoid conidium; Colony mor-phology of *Intumescentia ceratinae* sp. nov. (CGMCC3.23630) on PDA medium after 30 days at 25 °C, (**i**) top, (**j**) reverse, (**k**) asperulous hypha with intercalary swelling, (l) hypha with variable com-partment sizes, (**m**,**n**) lateral conidial initials, (**o**) chain of conidia with thick walls, (**p**) conidium. Bars = 5μm. Morphological structures listed above are indicated with arrows.

***Intumescentia pseudolivetorum*** H. L. Si, R. L. Chang, T. Bose and Y. C. Wang, sp. nov. (Figure 5a–h)

MycoBank No: 844859

Holotype: China, Yunnan Province: Tiesuo Township, 26°32′71” N, 100°57′3” E, ca. 2115 m elev., isolated from *Cetrelia pseudolivetorum*, 17 January 2021, H.L. Si, CX115A1a (HMAS 352172), ex-type Culture CGMCC3.23635, GenBank: ITS: OP345109, LSU: OP345119, SSU: OP345148, ACT: OP354479, CAL: OP354485, RPB2: OP354473, TEF1: OP354467. 

Etymology: Named after its host lichen species, *Cetrelia pseudolivetorum*.

Description: Hyphae are smooth, brown in color, septate, and the septa are slightly constricted, multi-guttulate, with columnar compartments that are variable in size, measuring 2.14–4.37 μm ($\bar{x}$ = 2.95 μm, n = 50), usually with sub-globose, pyriform, or irregular hyphal swelling that is apical or intercalary in position. Conidial cells are thick walled, catenulate, with two to eight in a chain, apical in position, and caducous, measuring 13.46–45.41 × 4.21–11.12 μm ($\bar{x}$= 24.5 × 5.99 μm, n = 11); the individual conidia are highly variable in shape, they can be bi- or tri-celled, septa transverse, fusoid to columnar in shape, brown in color, apical or lateral in position, measuring 2.94–15.7 × 3.03–7.07 μm ($\bar{x}$ = 6.65 × 4.4 μm, n = 50), and multi-guttulate. No sexual structure was observed.

Colony morphology and growth: Colony on PDA after 30 days at 25 °C is blackish brown in color (top and reverse), compact, superficial, tomentose, the margins are entire and irregularly lobed. Optimal growth temperature is 25 °C (0.27 mm/day). Growth was observed at 5 °C (0.06 mm/day), whereas no growth was detected at 35 °C.

Notes: *Intumescentia pseudolivetorum* emerged as the sister species to the clade that includes *I. tinctorum* and *I. vitii* (Figure 3). This species can be differentiated from other *Intumescentia* species based on the hyphal dimensions, position, shape, and measurement of the conidia (Table 1).

***Intumescentia ceratinae*** H. L. Si, R. L. Chang, T. Bose and Y. C. Wang, sp. nov. (Figure 5i–p)

MycoBank No: 844853

Holotype: China, Yunnan Province: Tiesuo Township, 26°32′71” N, 100°57′3” E, ca.2115 m elev., isolated from *Usnea ceratina*, 17 January 2021, H.L. Si, CX80A2 (HMAS 352147, Holotype), ex-type Culture CGMCC3.23630, GenBank: ITS: OP342838, LSU: OP345117, SSU: OP345112, ACT: OP354480, CAL: OP354486, RPB2: OP354474, TEF1: OP354468. 

Etymology: Named after its host lichen species, *Usnea ceratina*.

Description: Hyphae are asperulous, brown in color, septate, multi-guttulate, branched, compartments are variable in size, measuring 1.54–5.03 μm ($\bar{x}$ = 3.40 μm, n = 50), usually with globose, sub-globose, or irregular hyphal swelling that is apical or intercalary in position; lateral branching usually arises from swollen compartments. Conidial cells catenulate, with three to four in a chain, they are lateral or terminal in position, and caducous, measuring 7.16–75.41 × 2.09–4.93 μm ($\bar{x}$ = 31.61 × 3.5 μm, n = 50); each conidium is thick-walled, columnar in shape, dark brown in color, measuring 5.12–11.59 × 1.35–3.9 μm ($\bar{x}$ = 7.49 × 3.29 μm, n = 50), and multi-guttulate. Sexual structures were not observed. 

Colony morphology and growth: Colony on PDA after 30 days at 25 °C is blackish brown in color (top and reverse), compact, superficial, slightly raised in the center and villose, with margins that are finely serrated and irregularly lobed. Optimal growth temperature is 25 °C (0.4 mm/day). Growth was observed at 5 °C (0.06 mm/day), whereas no growth was detected at 35 °C. 

Notes: *Intumescentia ceratinae* has both unique and overlapping morphological characteristics with other *Intumescentia* species described in this study. The colony morphology, hyphal morphology, and conidia measurements distinguish this species from others, whereas the dimensions of the hyphal compartments, shape of the hyphal swellings, and optimal growth temperature of *I. ceratinae* are comparable to those of other *Intumescentia* species. In the phylogenetic analyses, *I. ceratinae* appeared as basal to other species in this family. However, this relationship did not receive significant branch support. Nonetheless, we placed this species in the genus *Intumescentia*. In the future, when more species from this genus are recovered, the taxonomic placement of this fungus can be re-evaluated. 

## 4. Discussion

For the present study, we conducted field surveys in Yunnan Province of China between 2020 and 2021. During these surveys, we collected specimens of 38 lichen species. From these lichens, we recovered 205 fungal isolates representing 127 fungal species from the phyla Ascomycota, Basidiomycota, and Zygomycota. This diversity included fungi from a wide variety of ecological guilds. Additionally, the fungal diversity overlapped between the lichen species and also included unique species. Among the fungi recovered in this study, there were six fungal isolates from the family Teratosphaeriaceae that had low morphological and genetic similarities with previously described species from this family. Multi-gene phylogenies indicated that these six isolates represented a novel clade within Teratosphaeriaceae. We erected a new genus *Intumescentia* to describe these six isolates as four new species: *I. ceratinae*, *I. vitii*, *I. wallichianae*, *I. tinctorum*, and *I. pseudolivetorum*.

In this study, 205 fungal isolates were identified from the phyla Ascomycota, Basidiomycota, and Mucoromycota. Among them, Dothideomycetes was the most dominant class (62 species), followed by Sordariomycetes (40 species), and Eurotiomycetes (13 species). The remaining species were from Agaricomycetes, Arthoniomycetes, Cystobasidiomycetes, Exobasidiomycetes, Leotiomycetes, Tremellomycetes, and Umbelopsidomycetes. Fungi from most of these classes have been previously reported from lichens [16]. However, the abundance of these classes substantially varies between the studies. For example, among the Antarctic lichens, Park et al. (2015) reported Arthoniomycetes, Eurotiomycetes, and Lecanoromycetes as the most common classes [54], whereas in a later study, Yu et al. (2018) found Leotiomycetes, Sordariomycetes, and Dothideomycetes to be abundant [22]. Similarly, Muggia et al. (2016) found that Chaetothyriomycetes and Dothideomycetes were the major fungal classes among rock-inhabiting lichens in the alpine area, whereas Rajulu et al. (2019) found Sordariomycetes, Dothideomycetes, and Eurotiomycetes to be prevalent among lichens in the Western Ghats, India [55,56]. The findings of these studies show that the environment, growth substrate, and a variety of other abiotic variables influence the fungal diversity associated with lichens. Simultaneously, the isolation and identification strategies used in many studies, including ours, can have an impact on the documented diversity.

In the present study, we recovered an assortment of fungi representing to a wide range of guilds, including saprophytes, plant pathogens, human pathogens, as well as entomopathogenic, endolichenic, and symbiotic fungi. This study supports the prior findings that fungal diversity associated with the lichen thallus is not limited to lichenicolous, endolichenic, and symbiotic fungi [16,57,58]. These lichen thalli also yielded human and entomopathogenic fungal species. However, we are uncertain of the specific ecological roles of these fungi in lichens. It is probable that these fungi perform additional roles in the environment than are currently understood. Alternatively, these fungi might have been contaminants during the isolation procedure. This hypothesis may be correct for fungi for which only a single isolate was recovered, but not for all.

Fungi from Teratosphaeriaceae are widely distributed. Several fungal species from this family have been identified from unique habitats. However, as of now, a few endolichenic fungi from Teratosphaeriaceae have been isolated from lichens [59], such as those from the genera *Xanthoriicola* and *Austrostigmidium* [12]. Contrarily, in a recent diversity study using a high throughput sequencing platform showed that Teratosphaeriaceae is the most abundant endolichenic fungal taxa associated with *Heterodermia obscurata* [13]. This contradiction is likely because a majority of endolichenic fungi are slow-growing, and in this regard, the isolates in this study are not an exception. Hence, it is difficult to isolate these fungi from a lichen thallus that houses a plethora of fungi. Still, it is important to conduct culture-based studies, such as this. Isolates recovered from this study and from other similar studies [26,27,60] will allow us to thoroughly investigate the biology of these fungi. In addition to this, some endolichenic fungi can also be of biotechnological importance, such as oleaginous yeasts [24].

For Teratosphaeriaceae, LSU is frequently used as the marker gene for identifying new fungal species [4,12,61,62,63]. This is because Crous et al. (2007) suggested LSU as a suitable marker for differentiating species within Teratosphaeriaceae [1]. However, there were insufficient variations in the LSU sequences between the six Teratosphaeriaceae fungal isolates recovered in this study. Nevertheless, amplifying the partial RPB2 gene allowed us to tease out the species. In the future, it is worth considering RPB2 along with LSU as markers for species delineation in Teratosphaeriaceae. The current study exemplifies the importance of culture-based fungal diversity studies from the lichen thallus and various other environments. In this era, a majority of the microbial diversity studies use one of the many available high-throughput sequencing platforms. This strategy is useful because it allows to identify a greater number of fungal OTUs along with many other advantages [64]. However, in most of the studies, the novel fungal species detected could not be resolved taxonomically. Additionally, now there is also provision for using sequences for describing novel fungi. However, this strategy should be used with caution, especially when using short-read sequencing platforms [65]. Even though culture-based diversity studies are currently rare, they allow for the recovery of fungal isolates that can feed into many downstream studies, such as taxonomy, genetics, and biotechnology. In the future, a hybrid approach using both of these strategies would be useful, such as those conducted for fungus-like organisms [66,67]. The data generated by these studies will be of interest to a broader scientific community and will not be limited to microbial diversity research groups.

## Figures and Tables

**Table 1:** Comparison of the morphological characters and growth rate of *Intumescentia* species recovered in this study.

	*Intumescentia* *tinctorum*	*Intumescentia* *vitii*	*Intumescentia* *pseudolivetorum*	*Intumescentia* *ceratinae*
**No. of isolates**	3	1	1	1
**Conidia**				
Characteristics	-	-	Thick walled, bi- or tri-celled, septa transverse, multi-guttulate	Thick-walled, dark brown in color, multi-guttulate
Shapes	-	-	Fusioid to columnar	Columnar to doliiform
Mean size (μm)	-	-	6.65 × 4.4	7.49 × 3.29
Range (μm)	-	-	2.94–15.7 × 3.03–7.07	5.12–11.59 × 1.35–3.9
**Chain of conidia**				
Characteristics	-	-	Catenulate, two to eight in a chain, apical in position, caducous,	Catenulate, three to four in a chain, lateral or terminal in position, caducous
Mean size (μm)	-	-	24.50 × 5.99	31.61 × 3.50
Range (μm)	-	-	13.46–45.41 × 4.21–11.12	7.16–75.41 × 2.09–4.93
**Sexual system**	Sterile	Sterile	Sterile	Sterile
**Hypha**				
Characteristics	Hyphae smooth, septate, multi-guttulate, branched, compartments variable in size and often distorted	Hyphae smooth, branched, septate, septa constricted, compartment often peanut-shaped, multi-guttulate, guttles small in size, compartment variable in size often distorted	Hyphae smooth, septate, septa slightly constricted, multi-guttulate, compartments columnar, variable in size	Hyphae asperulous, brown in color, septate, multi-guttulate, branched, compartments variable in size, usually with hyphal swelling that are apical or intercalary in position, lateral branching usually arising from swollen compartments.
Color	Light brown	Dark brown	Brown	Blackish brown
Mean size (μm)	3.23	4.09	2.95	3.4
Range (μm)	2.01–5.05	2.64–7.31	2.13–4.37	1.54–5.03
Hyphal swellings	Globose, sub-globose	Globose, irregular	Sub-globose, irregular	Globose, sub-globose, irregular
**Colony morphology on PDA**	Black-brown, compact, greenish gray in color, tomentose, margin entire, irregularly lobed	Blackish brown, compact, superficial, short tomentose, margin entire, irregularly lobed	Blackish brown, compact, superficial, tomentose, margin, entire, irregularly lobed	Blackish brown in colour, compact, superficial, slightly raised in the left and villose, margin finely serrated, irregularly lobed.
**Growth temperatures**				
Minimum (°C)	5	5	5	5
Maximum (°C)	30	30	30	30
Optimum (°C)	20	25	25	25

## Data Availability

All sequence data are available in NCBI GenBank following the accession numbers in the manuscript.

## Supplementary Materials

The following supporting information can be downloaded at: https://www.mdpi.com/article/10.3390/jof9040423/s1, Figure S1: Maximum likelihood phylogeny using ITS dataset for Teratosphaeriaceae. The bootstrap support value ≥75% and posterior probability ≥0.95 displayed above the node are ML/MP/PP. The isolates of *Intumescentia* gen. nov. obtained in this study are shown in bold. T = ex-type isolates; Figure S2: Maximum likelihood phylogeny using LSU dataset for Teratosphaeriaceae. The bootstrap support value ≥75% and posterior probability ≥0.95 displayed above the node are ML/MP/PP. The isolates of *Intumescentia* gen. nov. obtained in this study are shown in bold. T = ex-type isolates; Figure S3: Maximum likelihood phylogeny using TEF dataset for Teratosphaeriaceae. The bootstrap support value ≥75% and posterior probability ≥0.95 displayed above the node are ML/MP/PP. The isolates of *Intumescentia* gen. nov. obtained in this study are shown in bold. T = ex-type isolates; Figure S4: Maximum likelihood phylogeny using CAL dataset for Teratosphaeriaceae. The bootstrap support value ≥75% and posterior probability ≥0.95 displayed above the node are ML/MP/PP. The isolates of *Intumescentia* gen. nov. obtained in this study are shown in bold. T = ex-type isolates; Figure S5: Maximum likelihood phylogeny using RPB2 dataset for Teratosphaeriaceae. The bootstrap support value ≥75% and posterior probability ≥0.95 displayed above the node are ML/MP/PP. The isolates of *Intumescentia* gen. nov. obtained in this study are shown in bold. T = ex-type isolates; Figure S6: Maximum likelihood phylogeny using ACT dataset for Teratosphaeriaceae. The bootstrap support value ≥75% and posterior probability ≥0.95 displayed above the node are ML/MP/PP. The isolates of *Intumescentia* gen. nov. obtained in this study are shown in bold. T = ex-type isolates. Table S1: The lichen species sampled in this study, along with their sample numbers, habitat, distribution, and growth type; Table S2: Taxa used in the phylogenetic analyses and their corresponding GenBank numbers. T = ex-type isolates; Table S3: List of fungal species isolated from 38 lichen species sampled in this study and their respective guilds. Numbers in the cells indicate the number of isolates.

## References

[1] Crous P.W., Braun U., Groenewald J.Z. (2007). *Mycosphaerella* is polyphyletic. Stud. Mycol..

[2] Wijayawardene N.N. (2020). Outline of fungi and fungus-like taxa. Mycosphere.

[3] Wanasinghe D.N., Mortimer P.E., Xu J. (2021). Insight into the systematics of microfungi colonizing dead woody twigs of *Dodonaea viscosa* in Honghe (China). J. Fungi.

[4] Quaedvlieg W., Binder M., Groenewald J.Z., Summerell B.A., Carnegie A.J., Burgess T.I., Crous P.W. (2014). Introducing the consolidated species concept to resolve species in the teratosphaeriaceae. Persoonia.

[5] Barreto G., Gusmão L., Dianese J. (2022). Checklist of ascomycetes recorded on eucalypts in Brazil (1976–2022). Asian J. Mycol..

[6] Andjic V., Carnegie A.J., Pegg G.S., Hardy G.E.S.J., Maxwell A., Crous P.W., Pérez C., Wingfield M.J., Burgess T.I. (2019). 23 years of research on *Teratosphaeria* leaf blight of *Eucalyptus*. For. Ecol. Manage..

[7] Ruibal C., Selbmann L., Avci S., Martin-Sanchez P.M., Gorbushina A.A. (2018). Roof-Inhabiting cousins of rock-inhabiting fungi: Novel melanized microcolonial fungal species from photocatalytically reactive subaerial surfaces. Life.

[8] O’Connell L.M., Santos R., Springer G., Burne R.A., Nascimento M.M., Richards V.P. (2020). Site-specific profiling of the dental mycobiome reveals strong taxonomic shifts during progression of early-childhood caries. Appl. Environ. Microbiol..

[9] Coleine C., Pombubpa N., Zucconi L., Onofri S., Turchetti B., Buzzini P., Stajich J.E., Selbmann L. (2020). Uncovered microbial diversity in antarctic cryptoendolithic communities sampling three representative locations of the victoria land. Microorganisms.

[10] Morrison E.S., Thomas P., Ogram A., Kahveci T., Turner B.L., Chanton J.P. (2021). Characterization of bacterial and fungal communities reveals novel consortia in tropical oligotrophic peatlands. Microb. Ecol..

[11] Rizk S.M., Magdy M., Leo F., Werner O., Rashed M.A., Ros R.M., Urzi C. (2021). A new extremotolerant ecotype of the fungus *Pseudotaeniolina globosa* isolated from Djoser Pyramid, Memphis Necropolis, Egypt. J. Fungi.

[12] Pérez-Ortega S., Garrido-Benavent I., De Los Ríos A. (2015). *Austrostigmidium*, a new austral genus of lichenicolous fungi close to rock-inhabiting meristematic fungi in Teratosphaeriaceae. Lichenologist.

[13] Xu H., Wang L., Feng X., Gong X. (2022). Core taxa and photobiont-microbial interaction within the lichen *Heterodermia obscurata* (Physciaceae, Heterodermia). Symbiosis.

[14] Nash T.H. (2008). III Lichen Biology.

[15] Nelsen M.P., Lucking R., Boyce C.K., Lumbsch H.T., Ree R.H. (2020). No support for the emergence of lichens prior to the evolution of vascular plants. Geobiology.

[16] Muggia L., Grube M. (2018). Fungal diversity in lichens: From extremotolerance to interactions with algae. Life.

[17] Elkhateeb W., Daba G. (2021). Fungi over fungi, endophytic fungi associated with mushroom fruiting bodies and lichens. J. Pharm. Pharm. Res..

[18] Moreno L.F., Vicente V.A., de Hoog S. (2018). Black yeasts in the omics era: Achievements and challenges. Med. Mycol..

[19] Chakarwarti J. (2020). The diversity of endolichenic fungi—A review. Asian J. Mycol..

[20] U’Ren J.M., Lutzoni F., Miadlikowska J., Arnold A.E. (2010). Community analysis reveals close affinities between endophytic and endolichenic fungi in mosses and lichens. Microb. Ecol..

[21] Suryanarayanan T.S., Govindarajulu M.B., Rajamani T., Tripathi M., Joshi Y. (2017). Endolichenic fungi in lichens of Champawat district, Uttarakhand, northern India. Mycol. Prog..

[22] Yu N.H., Park S.Y., Kim J.A., Park C.H., Jeong M.H., Oh S.O., Hong S.G., Talavera M., Divakar P.K., Hur J.S. (2018). Endophytic and endolichenic fungal diversity in maritime Antarctica based on cultured material and their evolutionary position among Dikarya. Fungal Syst. Evol..

[23] Zhang T., Wei X.L., Wei Y.Z., Liu H.Y., Yu L.Y. (2016). Diversity and distribution of cultured endolichenic fungi in the Ny-Alesund Region, Svalbard (high arctic). Extremophiles.

[24] Chang R., Cao W., Wang Y., Li S., Li X., Bose T., Si H.L. (2022). *Melanodevriesia*, a new genus of endolichenic oleaginous black yeast recovered from the Inner Mongolia Region of China. Fungal Syst. Evol..

[25] Li W., Zhou J., Guo S., Guo L. (2007). Endophytic fungi associated with lichens in Baihua mountain of Beijing, China. Fungal Divers..

[26] Si H.L., Su Y.M., Zheng X.X., Ding M.Y., Bose T., Chang R.L. (2021). Phylogenetic and morphological analyses of *Coniochaeta* isolates recovered from Inner Mongolia and Yunnan revealed three new endolichenic fungal species. MycoKeys.

[27] Wang Q., Li J., Yang J., Zou Y., Zhao X.Q. (2022). Diversity of endophytic bacterial and fungal microbiota associated with the medicinal lichen *Usnea longissima* at high altitudes. Front. Microbiol..

[28] Si H.L., Zheng X.X., Lin X., Su Y.M., Bose T., Chang R.L. (2021). *Dlhawksworthia flavoparmeliae* sp. nov., a new endolichenic fungus in Phaeosphaeriaceae. Phytotaxa.

[29] Liu J., Hu Y., Luo X., Castañeda-Ruíz R.F., Xia J., Xu Z., Cui R., Shi X., Zhang L., Ma J. (2023). Molecular phylogeny and morphology reveal four novel species of *Corynespora* and *Kirschsteiniothelia* (Dothideomycetes, Ascomycota) from China: A checklist for *Corynespora* reported worldwide. J. Fungi.

[30] Wang L. (2012). Lichens of Yunnan in China.

[31] Wang L., Qian Z. (2012). Illustrated Medicinal Lichens of China.

[32] Gholibeigianet M. (2021). CTAB-Extraction method in Plant tissue. https://www.researchgate.net/publication/348693878..

[33] White T.J., Bruns T., Lee S., Taylor J., Innis M.A., Gelfand D.H., Sninsky J.J., White T.J. (1990). Amplification and direct sequencing of fungal ribosomal RNA genes for phylogenetics. PCR Protocols.

[34] Vilgalys R., Hester M. (1990). Rapid genetic identification and mapping of enzymatically amplified ribosomal DNA from several species of *Cryptococcus*. J. Bacteriol..

[35] Liu Y.J., Whelen S., Hall B.D. (1999). Phylogenetic relationships among ascomycetes: Evidence from an RNA polymerase II subunit. Mol. Biol. Evol..

[36] Groenewald J.Z., Nakashima C., Nishikawa J., Shin H.D., Park J.H., Jama A.N., Groenewald M., Braun U., Crous P.W. (2013). Species concepts in *Cercospora*: Spotting the weeds among the roses. Stud. Mycol..

[37] Carbone I., Kohn L.M. (1999). A method for designing primer sets for speciation studies in filamentous ascomycetes. Mycologia.

[38] Quaedvlieg W., Groenewald J.Z., de Jesús Yáñez-Morales M., Crous P.W. (2012). DNA barcoding of *Mycosphaerella* species of quarantine importance to Europe. Persoonia.

[39] Altschul S.F., Gish W., Miller W., Myers E.W., Lipman D.J. Basic local alignment search tool. J. Mol. Biol..

[40] Põlme S., Abarenkov K., Nilsson R.H., Lindahl B.D., Clemmensen K.E., Kauserud H., Nguyen N., Kjøller R., Bates S.T., Baldrian P. (2020). FungalTraits: A user-friendly traits database of fungi and fungus-like stramenopiles. Fungal Divers..

[41] Katoh K., Standley D. (2013). MAFFT multiple sequence alignment software version 7: Improvements in performance and usability. Mol. Biol. Evol..

[42] Sudhir K., Glen S., Koichiro T. (2016). MEGA7: Molecular evolutionary genetics analysis version 7.0 for bigger datasets. Mol. Biol. Evol..

[43] Miller M.A., Pfeiffer W., Schwartz T. Creating the CIPRES science gateway for inference of large phylogenetic trees. Proceedings of the Gateway Computing Environments Workshop (GCE).

[44] Darriba D., Taboada G.L., Doallo R., Posada D. (2012). jModelTest 2: More models, new heuristics and parallel computing. Nat. Methods.

[45] Stamatakis A., Hoover P., Rougemont J. (2008). A rapid bootstrap algorithm for the RAxML web servers. Syst. Biol..

[46] Ronquist F., Teslenko M., van der Mark P., Ayres D.L., Darling A., Hohna S., Larget B., Liu L., Suchard M.A., Huelsenbeck J.P. (2012). MrBayes 3.2: Efficient Bayesian phylogenetic inference and model choice across a large model space. Syst. Biol..

[47] Rambaut A. (2012). A Graphical Viewer of Phylogenetic Trees. http://tree.bio.ed.ac.uk/software/figtree/.

[48] Nirenberg H.I. (1976). Untersuchungen über die morphologische und biologische Differenzierung in der *Fusaium*-Sektion *Liseola*. Mitt. Biol. Bundesanst. Land-u. Forstwirtsch. Berlin Dahlem.

[49] Duarte A.P.M., Attili-Angelis D., Baron N.C., Groenewald J.Z., Crous P.W., Pagnocca F.C. (2017). Riding with the ants. Persoonia.

[50] Harrington A.H., Olmo-Ruiz M.d., U’Ren J.M., Garcia K., Pignatta D., Wespe N., Sandberg D.C., Huang Y.-L., Hoffman M.T., Arnold A.E. (2019). *Coniochaeta endophytica* sp. nov., a foliar endophyte associated with healthy photosynthetic tissue of *Platycladus orientalis* (cupressaceae). Plant Fungal Syst..

[51] Schneider C.A., Rasband W.S., Eliceiri K.W. (2012). NIH image to imageJ: 25 years of image analysis. Nat. Methods.

[52] Hujslová M., Kubátová A., Kostovčík M., Kolařík M. (2012). *Acidiella bohemica* gen. et sp. nov. and *Acidomyces* spp. (Teratosphaeriaceae), the indigenous inhabitants of extremely acidic soils in Europe. Fungal Divers..

[53] Crous P.W., Wingfield M.J., Burgess T.I., Hardy G., Gene J., Guarro J., Baseia I.G., Garcia D., Gusmao L.F.P., Souza-Motta C.M. (2018). Fungal planet description sheets: 716–784. Persoonia.

[54] Park C.H., Kim K.M., Elvebakk A., Kim O.-S., Jeong G., Hong S.G. (2015). Algal and fungal diversity in antarctic lichens. J. Eukaryot. Microbiol..

[55] Muggia L., Fleischhacker A., Kopun T., Grube M. (2016). Extremotolerant fungi from alpine rock lichens and their phylogenetic relationships. Fungal Divers..

[56] Rajulu M.B.G., Thirunavukkarasu N., Kumar S.S., Kaur T., Reddy M.S., Suryanarayanan T.S. (2019). Endolichenic fungal diversity associated with some lichens of the Western Ghats. Planta Med..

[57] Zhang T., Wei X.L., Zhang Y.Q., Liu H.Y., Yu L.Y. (2015). Diversity and distribution of lichen-associated fungi in the Ny-Alesund region (Svalbard, High Arctic) as revealed by 454 pyrosequencing. Sci. Rep..

[58] Petrini O., Hake U., Dreyfuss M.M. (1990). An analysis of fungal communities isolated from fruticose lichens. Mycologia.

[59] Diederich P., Lawrey J.D., Ertz D. (2018). The 2018 classification and checklist of lichenicolous fungi, with 2000 non-lichenized, obligately lichenicolous taxa. Bryologist.

[60] Kannangara B.T., Rajapaksha R.S., Paranagama P.A. (2009). Nature and bioactivities of endolichenic fungi in *Pseudocyphellaria* sp., *Parmotrema* sp. and *Usnea* sp. at Hakgala montane forest in Sri Lanka. Lett. Appl. Microbiol..

[61] Chen C.-H., Hsieh S.-Y., Yeh Y.-H., Kirschner R. (2020). *Cladocillium musae*, a new genus and species of cercosporoid fungi (Mycosphaerellaceae) on wild banana in Taiwan. Mycol. Prog..

[62] Flakus A., Etayo J., Perez-Ortega S., Kukwa M., Palice Z., Rodriguez-Flakus P. (2019). A new genus, *Zhurbenkoa*, and a novel nutritional mode revealed in the family Malmideaceae (Lecanoromycetes, Ascomycota). Mycologia.

[63] Meswaet Y., Mangelsdorff R., Yorou N.S., Piepenbring M. (2021). Unravelling unexplored diversity of cercosporoid fungi (mycosphaerellaceae, mycosphaerellales, ascomycota) in tropical Africa. MycoKeys.

[64] Baldrian P., Větrovský T., Lepinay C., Kohout P. (2021). High-throughput sequencing view on the magnitude of global fungal diversity. Fungal Divers..

[65] Thines M., Crous P.W., Aime M.C., Aoki T., Cai L., Hyde K.D., Miller A.N., Zhang N., Stadler M. (2018). Ten reasons why a sequence-based nomenclature is not useful for fungi anytime soon. IMA Fungus.

[66] Legeay J., Husson C., Boudier B., Louisanna E., Baraloto C., Schimann H., Marcais B., Buee M. (2020). Surprising low diversity of the plant pathogen *Phytophthora* in Amazonian forests. Environ. Microbiol..

[67] Bose T., Wingfield M.J., Roux J., Vivas M., Burgess T.I. (2018). Community composition and distribution of *Phytophthora* species across adjacent native and non-native forests of South Africa. Fungal Ecol..

