# Supplementary material for: Fungal Diversity Associated with Thirty-Eight Lichen Species Revealed a New Genus of Endolichenic Fungi, Intumescentia gen. nov. (Teratosphaeriaceae)
Source: J Fungi (Basel). 2023 Mar 29;9(4):423. doi: 10.3390/jof9040423 (PMC10143819; doi:10.3390/jof9040423)
Supplement: Supplementary file 1 [file jof-09-00423-s001.zip › Figure S1 ITS.pptx]

## Slide 1
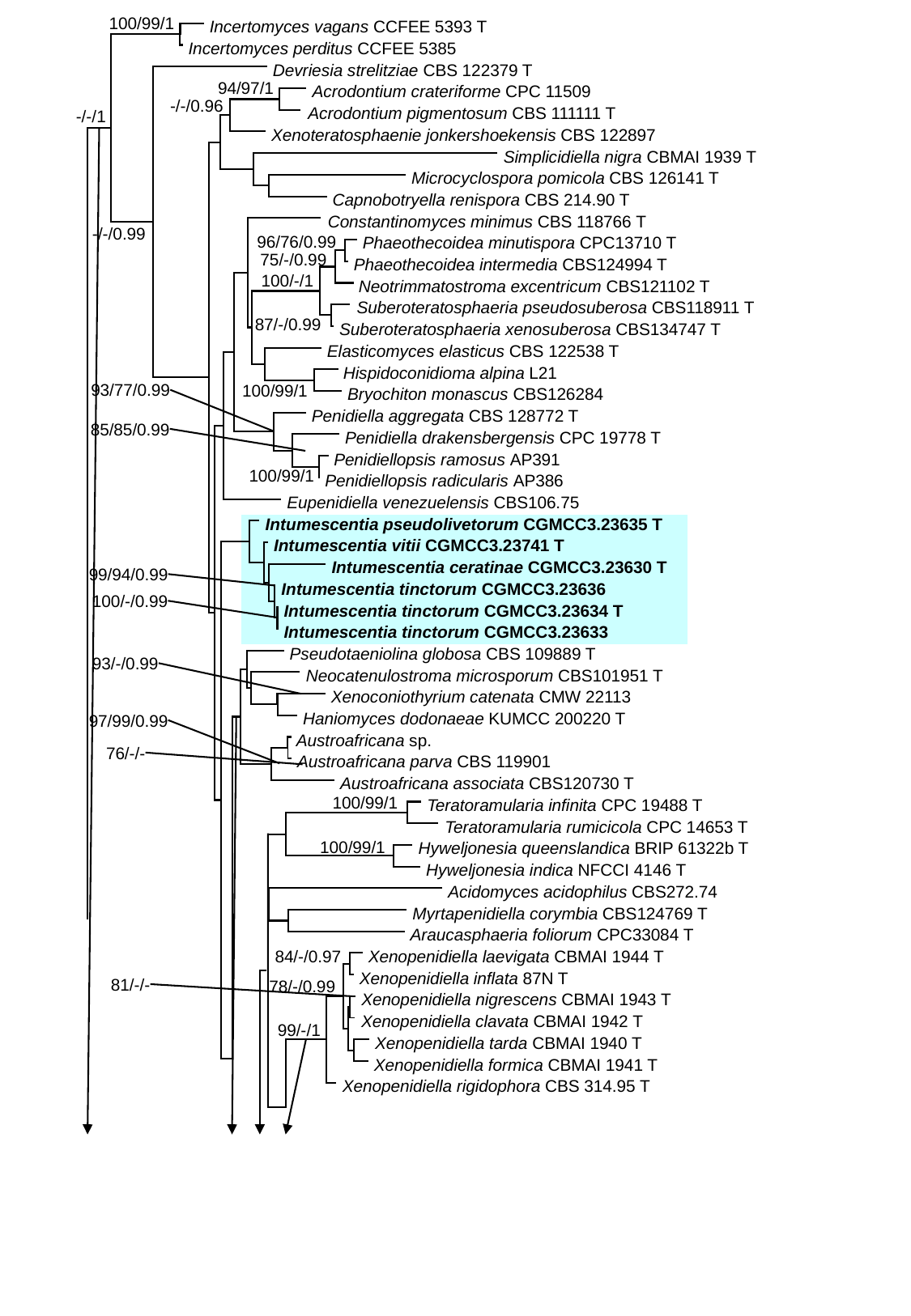

100/99/1
 Incertomyces vagans CCFEE 5393 T
 Incertomyces perditus CCFEE 5385
 Devriesia strelitziae CBS 122379 T
 Acrodontium crateriforme CPC 11509
 Acrodontium pigmentosum CBS 111111 T
 Xenoteratosphaenie jonkershoekensis CBS 122897
 Simplicidiella nigra CBMAI 1939 T
 Microcyclospora pomicola CBS 126141 T
 Capnobotryella renispora CBS 214.90 T
 Constantinomyces minimus CBS 118766 T
 Phaeothecoidea minutispora CPC13710 T
 Phaeothecoidea intermedia CBS124994 T
 Neotrimmatostroma excentricum CBS121102 T
 Suberoteratosphaeria pseudosuberosa CBS118911 T
 Suberoteratosphaeria xenosuberosa CBS134747 T
 Elasticomyces elasticus CBS 122538 T
 Hispidoconidioma alpina L21
 Bryochiton monascus CBS126284
 Penidiella aggregata CBS 128772 T
 Penidiella drakensbergensis CPC 19778 T
 Penidiellopsis ramosus AP391
 Penidiellopsis radicularis AP386
 Eupenidiella venezuelensis CBS106.75
 Intumescentia pseudolivetorum CGMCC3.23635 T
 Intumescentia vitii CGMCC3.23741 T
 Intumescentia ceratinae CGMCC3.23630 T
 Intumescentia tinctorum CGMCC3.23636
 Intumescentia tinctorum CGMCC3.23634 T
 Intumescentia tinctorum CGMCC3.23633
 Pseudotaeniolina globosa CBS 109889 T
 Neocatenulostroma microsporum CBS101951 T
 Xenoconiothyrium catenata CMW 22113
 Haniomyces dodonaeae KUMCC 200220 T
 Austroafricana sp.
 Austroafricana parva CBS 119901
 Austroafricana associata CBS120730 T
 Teratoramularia infinita CPC 19488 T
 Teratoramularia rumicicola CPC 14653 T
 Hyweljonesia queenslandica BRIP 61322b T
 Hyweljonesia indica NFCCI 4146 T
 Acidomyces acidophilus CBS272.74
 Myrtapenidiella corymbia CBS124769 T
 Araucasphaeria foliorum CPC33084 T
 Xenopenidiella laevigata CBMAI 1944 T
 Xenopenidiella inflata 87N T
 Xenopenidiella nigrescens CBMAI 1943 T
 Xenopenidiella clavata CBMAI 1942 T
 Xenopenidiella tarda CBMAI 1940 T
 Xenopenidiella formica CBMAI 1941 T
 Xenopenidiella rigidophora CBS 314.95 T
94/97/1
-/-/0.96
-/-/1
-/-/0.99
96/76/0.99
75/-/0.99
100/-/1
87/-/0.99
93/77/0.99
100/99/1
85/85/0.99
100/99/1
99/94/0.99
100/-/0.99
93/-/0.99
97/99/0.99
76/-/-
100/99/1
100/99/1
84/-/0.97
81/-/-
78/-/0.99
99/-/1

## Slide 2
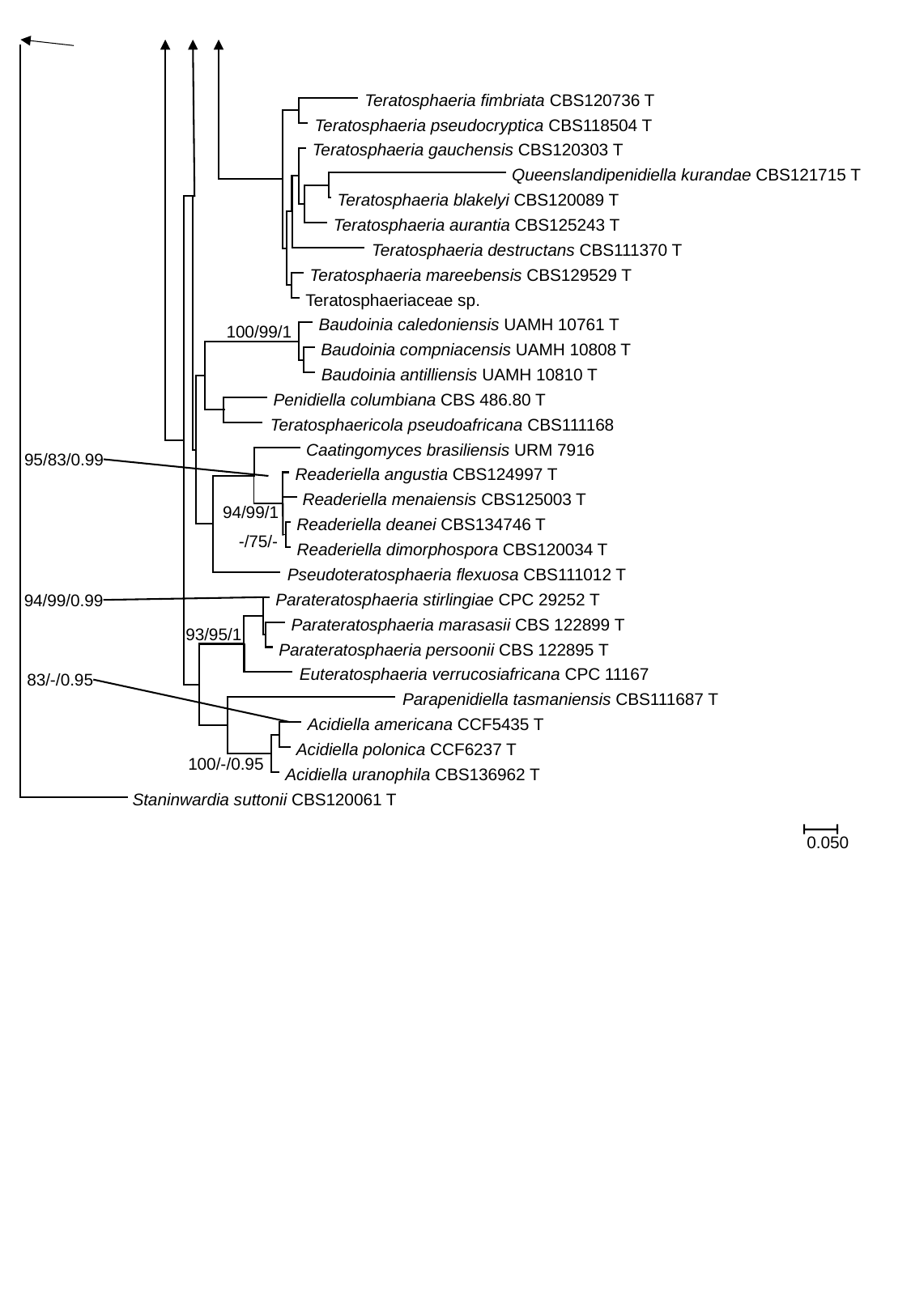

Teratosphaeria fimbriata CBS120736 T
 Teratosphaeria pseudocryptica CBS118504 T
 Teratosphaeria gauchensis CBS120303 T
 Queenslandipenidiella kurandae CBS121715 T
 Teratosphaeria blakelyi CBS120089 T
 Teratosphaeria aurantia CBS125243 T
 Teratosphaeria destructans CBS111370 T
 Teratosphaeria mareebensis CBS129529 T
 Teratosphaeriaceae sp.
 Baudoinia caledoniensis UAMH 10761 T
 Baudoinia compniacensis UAMH 10808 T
 Baudoinia antilliensis UAMH 10810 T
 Penidiella columbiana CBS 486.80 T
 Teratosphaericola pseudoafricana CBS111168
 Caatingomyces brasiliensis URM 7916
 Readeriella angustia CBS124997 T
 Readeriella menaiensis CBS125003 T
 Readeriella deanei CBS134746 T
 Readeriella dimorphospora CBS120034 T
 Pseudoteratosphaeria flexuosa CBS111012 T
100/99/1
95/83/0.99
94/99/1
-/75/-
 Parateratosphaeria stirlingiae CPC 29252 T
94/99/0.99
 Parateratosphaeria marasasii CBS 122899 T
93/95/1
 Parateratosphaeria persoonii CBS 122895 T
 Euteratosphaeria verrucosiafricana CPC 11167
83/-/0.95
 Parapenidiella tasmaniensis CBS111687 T
 Acidiella americana CCF5435 T
 Acidiella polonica CCF6237 T
100/-/0.95
 Acidiella uranophila CBS136962 T
 Staninwardia suttonii CBS120061 T
0.050
