# Supplementary material for: Fungal Diversity Associated with Thirty-Eight Lichen Species Revealed a New Genus of Endolichenic Fungi, Intumescentia gen. nov. (Teratosphaeriaceae)
Source: J Fungi (Basel). 2023 Mar 29;9(4):423. doi: 10.3390/jof9040423 (PMC10143819; doi:10.3390/jof9040423)
Supplement: Supplementary file 1 [file jof-09-00423-s001.zip › Figure S2 LSU.pptx]

## Slide 1
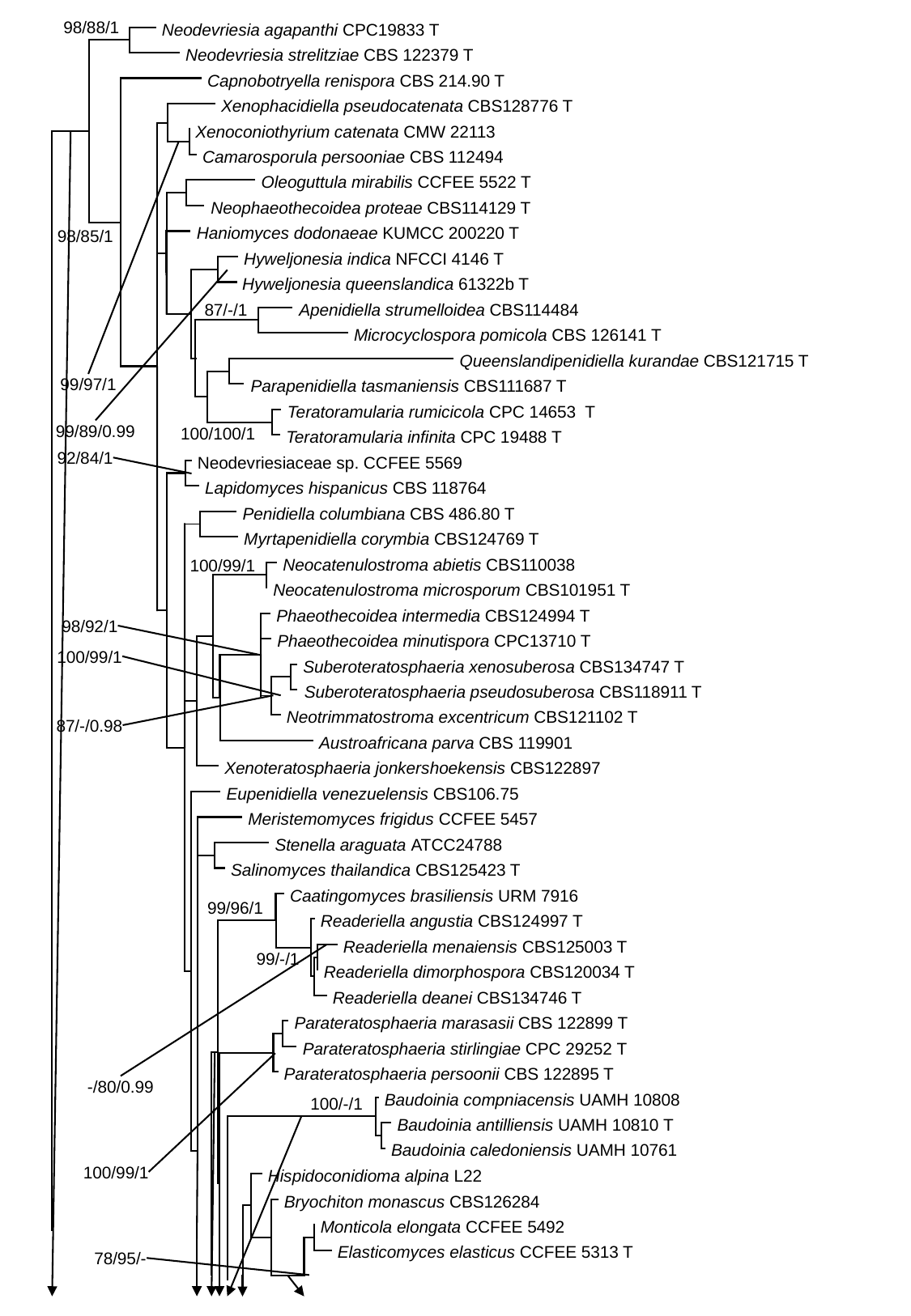

98/88/1
 Neodevriesia agapanthi CPC19833 T
 Neodevriesia strelitziae CBS 122379 T
 Capnobotryella renispora CBS 214.90 T
 Xenophacidiella pseudocatenata CBS128776 T
 Xenoconiothyrium catenata CMW 22113
 Camarosporula persooniae CBS 112494
 Oleoguttula mirabilis CCFEE 5522 T
 Neophaeothecoidea proteae CBS114129 T
 Haniomyces dodonaeae KUMCC 200220 T
 Hyweljonesia indica NFCCI 4146 T
 Hyweljonesia queenslandica 61322b T
 Apenidiella strumelloidea CBS114484
 Microcyclospora pomicola CBS 126141 T
 Queenslandipenidiella kurandae CBS121715 T
 Parapenidiella tasmaniensis CBS111687 T
 Teratoramularia rumicicola CPC 14653 T
 Teratoramularia infinita CPC 19488 T
 Neodevriesiaceae sp. CCFEE 5569
 Lapidomyces hispanicus CBS 118764
 Penidiella columbiana CBS 486.80 T
 Myrtapenidiella corymbia CBS124769 T
 Neocatenulostroma abietis CBS110038
 Neocatenulostroma microsporum CBS101951 T
 Phaeothecoidea intermedia CBS124994 T
 Phaeothecoidea minutispora CPC13710 T
 Suberoteratosphaeria xenosuberosa CBS134747 T
 Suberoteratosphaeria pseudosuberosa CBS118911 T
 Neotrimmatostroma excentricum CBS121102 T
 Austroafricana parva CBS 119901
 Xenoteratosphaeria jonkershoekensis CBS122897
 Eupenidiella venezuelensis CBS106.75
 Meristemomyces frigidus CCFEE 5457
 Stenella araguata ATCC24788
 Salinomyces thailandica CBS125423 T
 Caatingomyces brasiliensis URM 7916
 Readeriella angustia CBS124997 T
 Readeriella menaiensis CBS125003 T
 Readeriella dimorphospora CBS120034 T
 Readeriella deanei CBS134746 T
 Parateratosphaeria marasasii CBS 122899 T
 Parateratosphaeria stirlingiae CPC 29252 T
 Parateratosphaeria persoonii CBS 122895 T
 Baudoinia compniacensis UAMH 10808
 Baudoinia antilliensis UAMH 10810 T
 Baudoinia caledoniensis UAMH 10761
 Hispidoconidioma alpina L22
 Bryochiton monascus CBS126284
 Monticola elongata CCFEE 5492
 Elasticomyces elasticus CCFEE 5313 T
98/85/1
87/-/1
99/97/1
99/89/0.99
100/100/1
92/84/1
100/99/1
98/92/1
100/99/1
87/-/0.98
99/96/1
99/-/1
-/80/0.99
100/-/1
100/99/1
78/95/-

## Slide 2
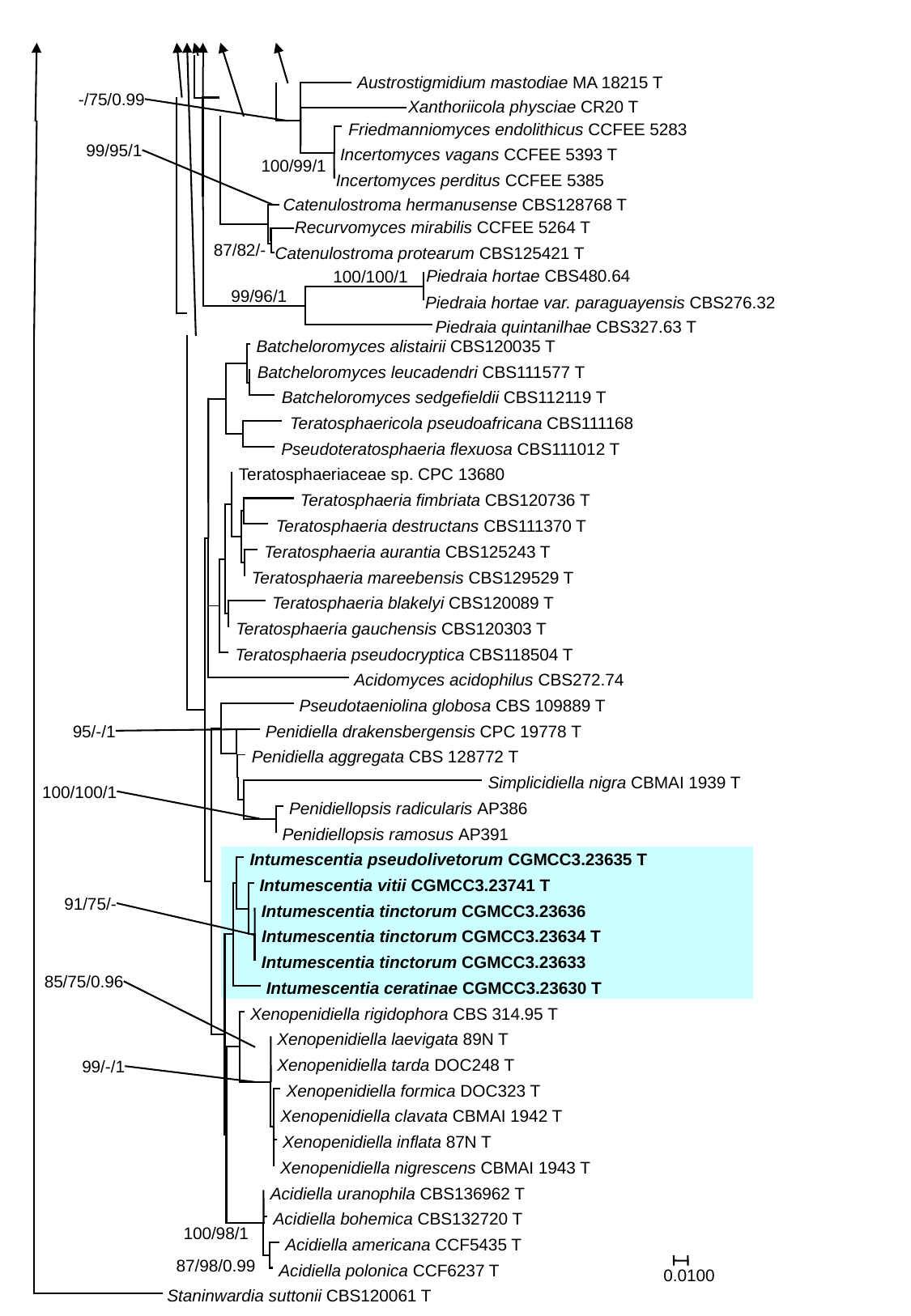

Austrostigmidium mastodiae MA 18215 T
-/75/0.99
 Xanthoriicola physciae CR20 T
 Friedmanniomyces endolithicus CCFEE 5283
99/95/1
 Incertomyces vagans CCFEE 5393 T
100/99/1
 Incertomyces perditus CCFEE 5385
 Catenulostroma hermanusense CBS128768 T
 Recurvomyces mirabilis CCFEE 5264 T
87/82/-
 Catenulostroma protearum CBS125421 T
 Piedraia hortae CBS480.64
100/100/1
99/96/1
 Piedraia hortae var. paraguayensis CBS276.32
 Piedraia quintanilhae CBS327.63 T
 Batcheloromyces alistairii CBS120035 T
 Batcheloromyces leucadendri CBS111577 T
 Batcheloromyces sedgefieldii CBS112119 T
 Teratosphaericola pseudoafricana CBS111168
 Pseudoteratosphaeria flexuosa CBS111012 T
 Teratosphaeriaceae sp. CPC 13680
 Teratosphaeria fimbriata CBS120736 T
 Teratosphaeria destructans CBS111370 T
 Teratosphaeria aurantia CBS125243 T
 Teratosphaeria mareebensis CBS129529 T
 Teratosphaeria blakelyi CBS120089 T
 Teratosphaeria gauchensis CBS120303 T
 Teratosphaeria pseudocryptica CBS118504 T
 Acidomyces acidophilus CBS272.74
 Pseudotaeniolina globosa CBS 109889 T
95/-/1
 Penidiella drakensbergensis CPC 19778 T
 Penidiella aggregata CBS 128772 T
 Simplicidiella nigra CBMAI 1939 T
100/100/1
 Penidiellopsis radicularis AP386
 Penidiellopsis ramosus AP391
 Intumescentia pseudolivetorum CGMCC3.23635 T
 Intumescentia vitii CGMCC3.23741 T
91/75/-
 Intumescentia tinctorum CGMCC3.23636
 Intumescentia tinctorum CGMCC3.23634 T
 Intumescentia tinctorum CGMCC3.23633
85/75/0.96
 Intumescentia ceratinae CGMCC3.23630 T
 Xenopenidiella rigidophora CBS 314.95 T
 Xenopenidiella laevigata 89N T
 Xenopenidiella tarda DOC248 T
99/-/1
 Xenopenidiella formica DOC323 T
 Xenopenidiella clavata CBMAI 1942 T
 Xenopenidiella inflata 87N T
 Xenopenidiella nigrescens CBMAI 1943 T
 Acidiella uranophila CBS136962 T
 Acidiella bohemica CBS132720 T
100/98/1
 Acidiella americana CCF5435 T
87/98/0.99
 Acidiella polonica CCF6237 T
0.0100
 Staninwardia suttonii CBS120061 T
