# Supplementary material for: Fungal Diversity Associated with Thirty-Eight Lichen Species Revealed a New Genus of Endolichenic Fungi, Intumescentia gen. nov. (Teratosphaeriaceae)
Source: J Fungi (Basel). 2023 Mar 29;9(4):423. doi: 10.3390/jof9040423 (PMC10143819; doi:10.3390/jof9040423)
Supplement: Supplementary file 1 [file jof-09-00423-s001.zip › Figure S3 TEF.pptx]

## Slide 1
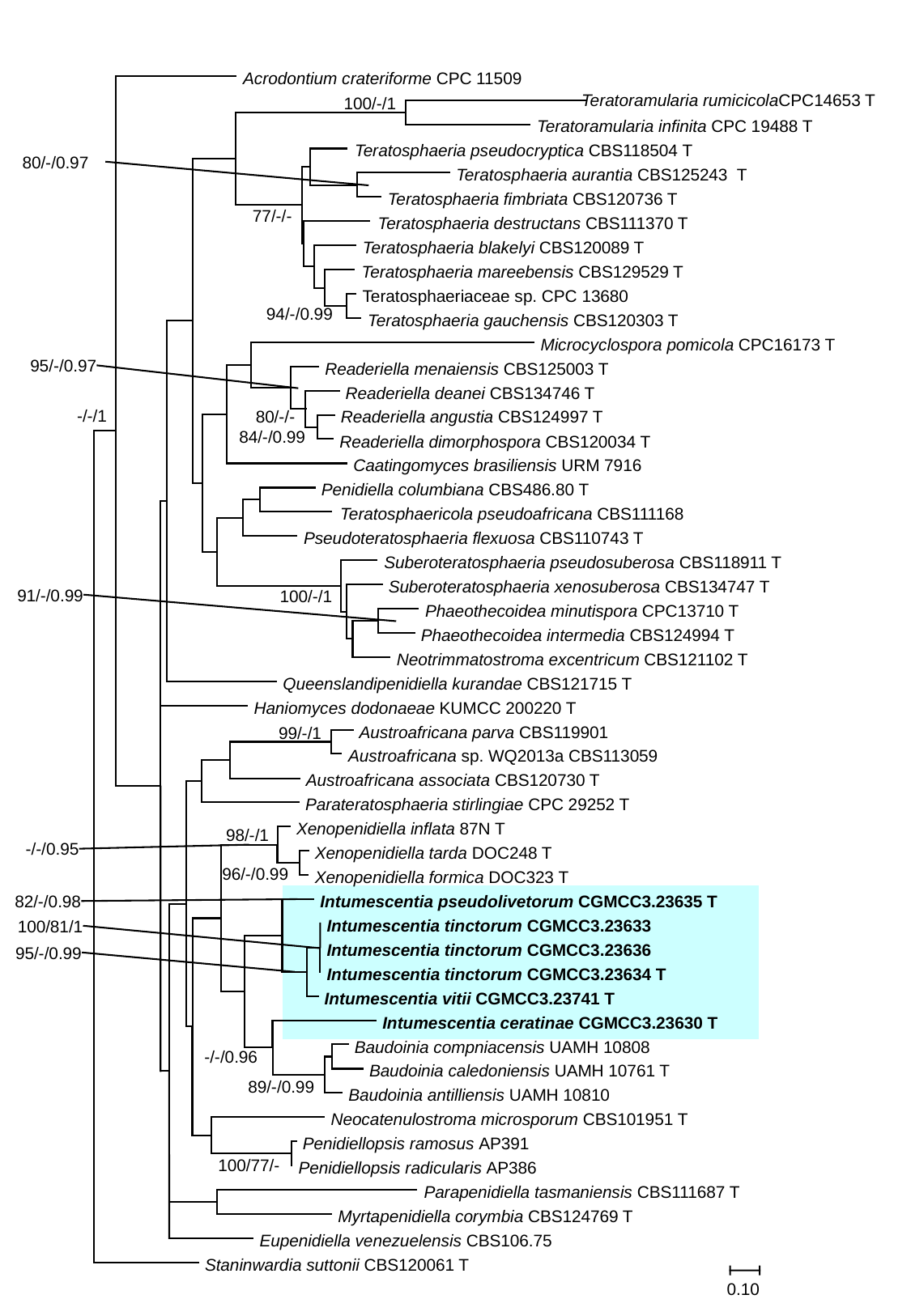

Acrodontium crateriforme CPC 11509
 Teratoramularia rumicicolaCPC14653 T
100/-/1
 Teratoramularia infinita CPC 19488 T
 Teratosphaeria pseudocryptica CBS118504 T
80/-/0.97
 Teratosphaeria aurantia CBS125243 T
 Teratosphaeria fimbriata CBS120736 T
77/-/-
 Teratosphaeria destructans CBS111370 T
 Teratosphaeria blakelyi CBS120089 T
 Teratosphaeria mareebensis CBS129529 T
 Teratosphaeriaceae sp. CPC 13680
94/-/0.99
 Teratosphaeria gauchensis CBS120303 T
 Microcyclospora pomicola CPC16173 T
95/-/0.97
 Readeriella menaiensis CBS125003 T
 Readeriella deanei CBS134746 T
-/-/1
80/-/-
 Readeriella angustia CBS124997 T
84/-/0.99
 Readeriella dimorphospora CBS120034 T
 Caatingomyces brasiliensis URM 7916
 Penidiella columbiana CBS486.80 T
 Teratosphaericola pseudoafricana CBS111168
 Pseudoteratosphaeria flexuosa CBS110743 T
 Suberoteratosphaeria pseudosuberosa CBS118911 T
 Suberoteratosphaeria xenosuberosa CBS134747 T
91/-/0.99
100/-/1
 Phaeothecoidea minutispora CPC13710 T
 Phaeothecoidea intermedia CBS124994 T
 Neotrimmatostroma excentricum CBS121102 T
 Queenslandipenidiella kurandae CBS121715 T
 Haniomyces dodonaeae KUMCC 200220 T
 Austroafricana parva CBS119901
99/-/1
 Austroafricana sp. WQ2013a CBS113059
 Austroafricana associata CBS120730 T
 Parateratosphaeria stirlingiae CPC 29252 T
 Xenopenidiella inflata 87N T
98/-/1
-/-/0.95
 Xenopenidiella tarda DOC248 T
96/-/0.99
 Xenopenidiella formica DOC323 T
82/-/0.98
 Intumescentia pseudolivetorum CGMCC3.23635 T
 Intumescentia tinctorum CGMCC3.23633
100/81/1
 Intumescentia tinctorum CGMCC3.23636
95/-/0.99
 Intumescentia tinctorum CGMCC3.23634 T
 Intumescentia vitii CGMCC3.23741 T
 Intumescentia ceratinae CGMCC3.23630 T
 Baudoinia compniacensis UAMH 10808
-/-/0.96
 Baudoinia caledoniensis UAMH 10761 T
89/-/0.99
 Baudoinia antilliensis UAMH 10810
 Neocatenulostroma microsporum CBS101951 T
 Penidiellopsis ramosus AP391
100/77/-
 Penidiellopsis radicularis AP386
 Parapenidiella tasmaniensis CBS111687 T
 Myrtapenidiella corymbia CBS124769 T
 Eupenidiella venezuelensis CBS106.75
 Staninwardia suttonii CBS120061 T
0.10
