# Supplementary material for: Fungal Diversity Associated with Thirty-Eight Lichen Species Revealed a New Genus of Endolichenic Fungi, Intumescentia gen. nov. (Teratosphaeriaceae)
Source: J Fungi (Basel). 2023 Mar 29;9(4):423. doi: 10.3390/jof9040423 (PMC10143819; doi:10.3390/jof9040423)
Supplement: Supplementary file 1 [file jof-09-00423-s001.zip › Figure S4 CAL.pptx]

## Slide 1
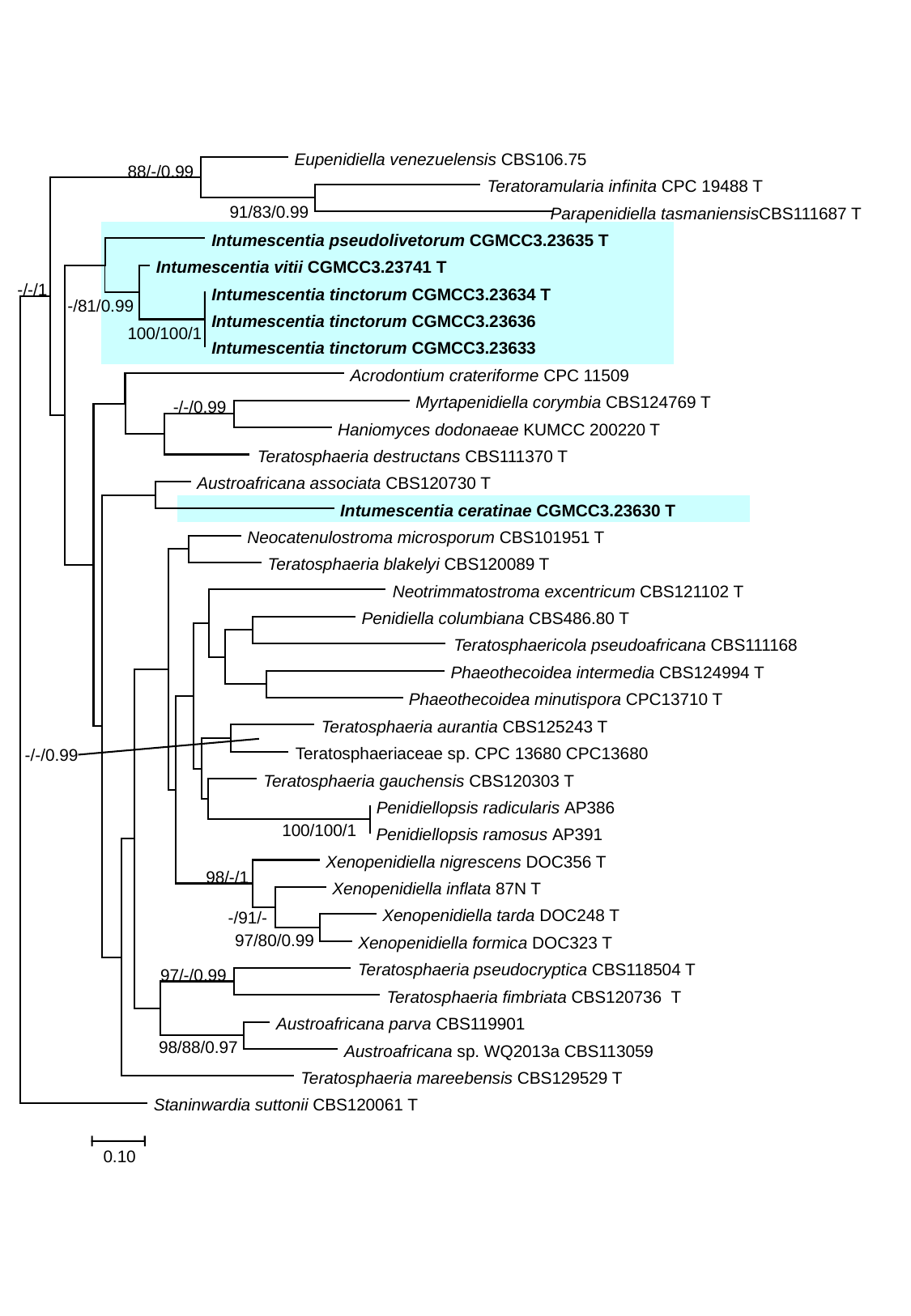

Eupenidiella venezuelensis CBS106.75
88/-/0.99
 Teratoramularia infinita CPC 19488 T
91/83/0.99
 Parapenidiella tasmaniensisCBS111687 T
 Intumescentia pseudolivetorum CGMCC3.23635 T
 Intumescentia vitii CGMCC3.23741 T
-/-/1
 Intumescentia tinctorum CGMCC3.23634 T
-/81/0.99
 Intumescentia tinctorum CGMCC3.23636
100/100/1
 Intumescentia tinctorum CGMCC3.23633
 Acrodontium crateriforme CPC 11509
 Myrtapenidiella corymbia CBS124769 T
-/-/0.99
 Haniomyces dodonaeae KUMCC 200220 T
 Teratosphaeria destructans CBS111370 T
 Austroafricana associata CBS120730 T
 Intumescentia ceratinae CGMCC3.23630 T
 Neocatenulostroma microsporum CBS101951 T
 Teratosphaeria blakelyi CBS120089 T
 Neotrimmatostroma excentricum CBS121102 T
 Penidiella columbiana CBS486.80 T
 Teratosphaericola pseudoafricana CBS111168
 Phaeothecoidea intermedia CBS124994 T
 Phaeothecoidea minutispora CPC13710 T
 Teratosphaeria aurantia CBS125243 T
 Teratosphaeriaceae sp. CPC 13680 CPC13680
-/-/0.99
 Teratosphaeria gauchensis CBS120303 T
 Penidiellopsis radicularis AP386
100/100/1
 Penidiellopsis ramosus AP391
 Xenopenidiella nigrescens DOC356 T
98/-/1
 Xenopenidiella inflata 87N T
 Xenopenidiella tarda DOC248 T
-/91/-
97/80/0.99
 Xenopenidiella formica DOC323 T
 Teratosphaeria pseudocryptica CBS118504 T
97/-/0.99
 Teratosphaeria fimbriata CBS120736 T
 Austroafricana parva CBS119901
98/88/0.97
 Austroafricana sp. WQ2013a CBS113059
 Teratosphaeria mareebensis CBS129529 T
 Staninwardia suttonii CBS120061 T
0.10
