# Supplementary material for: Fungal Diversity Associated with Thirty-Eight Lichen Species Revealed a New Genus of Endolichenic Fungi, Intumescentia gen. nov. (Teratosphaeriaceae)
Source: J Fungi (Basel). 2023 Mar 29;9(4):423. doi: 10.3390/jof9040423 (PMC10143819; doi:10.3390/jof9040423)
Supplement: Supplementary file 1 [file jof-09-00423-s001.zip › Figure S5 RPB2.pptx]

## Slide 1
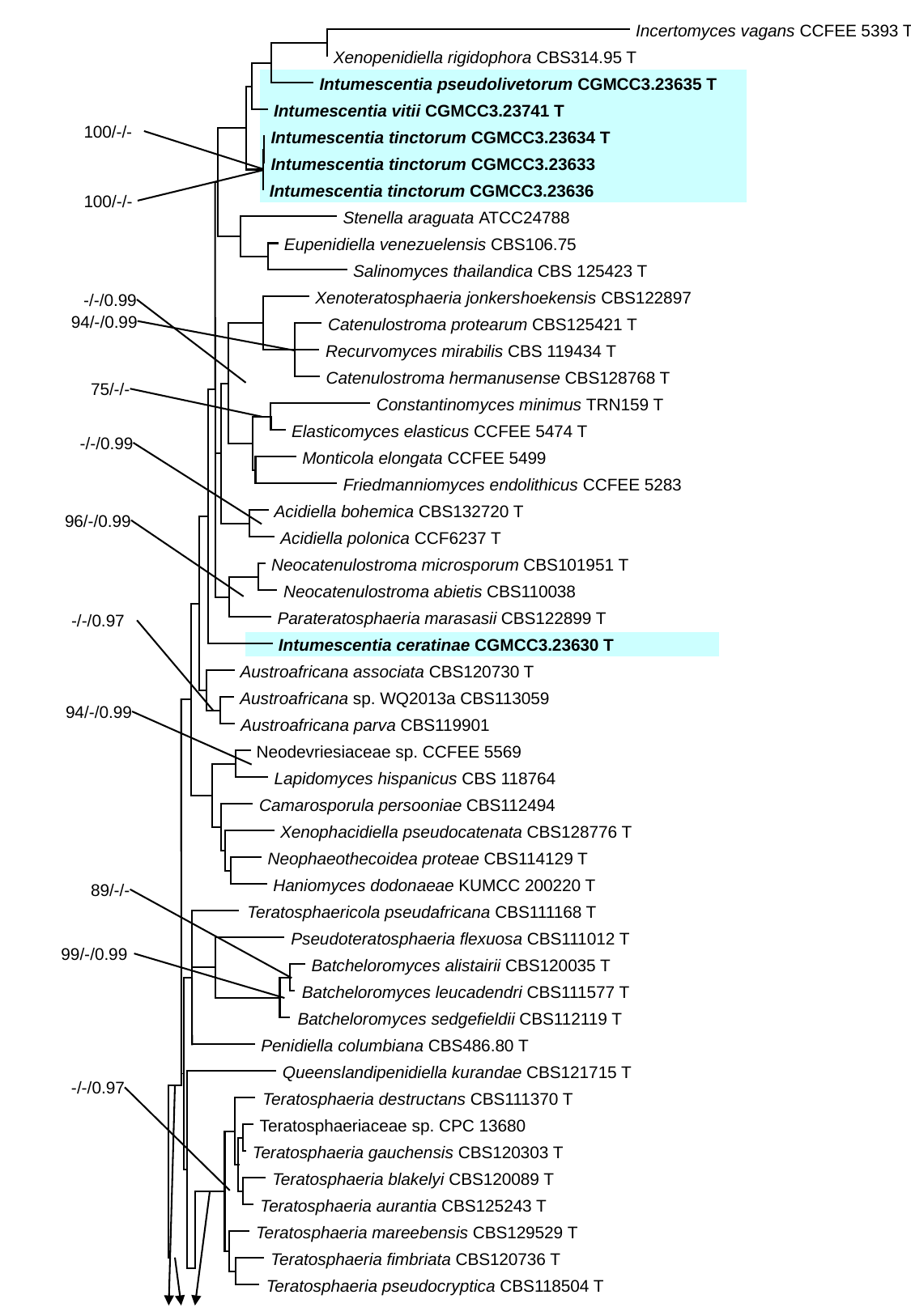

Incertomyces vagans CCFEE 5393 T
 Xenopenidiella rigidophora CBS314.95 T
 Intumescentia pseudolivetorum CGMCC3.23635 T
 Intumescentia vitii CGMCC3.23741 T
 Intumescentia tinctorum CGMCC3.23634 T
 Intumescentia tinctorum CGMCC3.23633
 Intumescentia tinctorum CGMCC3.23636
 Stenella araguata ATCC24788
 Eupenidiella venezuelensis CBS106.75
 Salinomyces thailandica CBS 125423 T
 Xenoteratosphaeria jonkershoekensis CBS122897
 Catenulostroma protearum CBS125421 T
 Recurvomyces mirabilis CBS 119434 T
 Catenulostroma hermanusense CBS128768 T
 Constantinomyces minimus TRN159 T
 Elasticomyces elasticus CCFEE 5474 T
 Monticola elongata CCFEE 5499
 Friedmanniomyces endolithicus CCFEE 5283
 Acidiella bohemica CBS132720 T
 Acidiella polonica CCF6237 T
 Neocatenulostroma microsporum CBS101951 T
 Neocatenulostroma abietis CBS110038
 Parateratosphaeria marasasii CBS122899 T
 Intumescentia ceratinae CGMCC3.23630 T
 Austroafricana associata CBS120730 T
 Austroafricana sp. WQ2013a CBS113059
 Austroafricana parva CBS119901
 Neodevriesiaceae sp. CCFEE 5569
 Lapidomyces hispanicus CBS 118764
 Camarosporula persooniae CBS112494
 Xenophacidiella pseudocatenata CBS128776 T
 Neophaeothecoidea proteae CBS114129 T
 Haniomyces dodonaeae KUMCC 200220 T
 Teratosphaericola pseudafricana CBS111168 T
 Pseudoteratosphaeria flexuosa CBS111012 T
 Batcheloromyces alistairii CBS120035 T
 Batcheloromyces leucadendri CBS111577 T
 Batcheloromyces sedgefieldii CBS112119 T
 Penidiella columbiana CBS486.80 T
 Queenslandipenidiella kurandae CBS121715 T
 Teratosphaeria destructans CBS111370 T
 Teratosphaeriaceae sp. CPC 13680
 Teratosphaeria gauchensis CBS120303 T
 Teratosphaeria blakelyi CBS120089 T
 Teratosphaeria aurantia CBS125243 T
 Teratosphaeria mareebensis CBS129529 T
 Teratosphaeria fimbriata CBS120736 T
 Teratosphaeria pseudocryptica CBS118504 T
100/-/-
100/-/-
-/-/0.99
94/-/0.99
75/-/-
-/-/0.99
96/-/0.99
-/-/0.97
94/-/0.99
89/-/-
99/-/0.99
-/-/0.97

## Slide 2
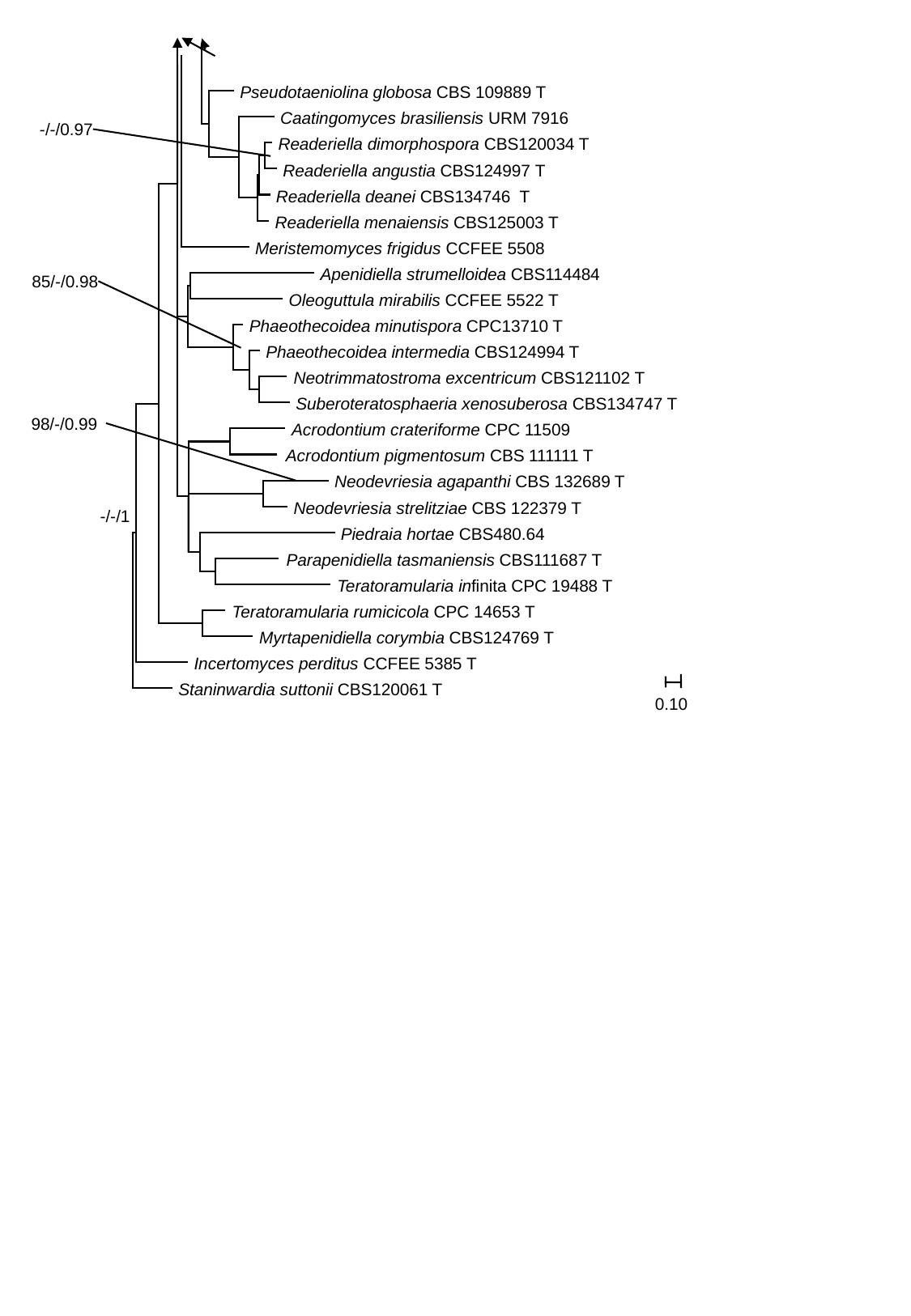

Pseudotaeniolina globosa CBS 109889 T
 Caatingomyces brasiliensis URM 7916
 Readeriella dimorphospora CBS120034 T
 Readeriella angustia CBS124997 T
 Readeriella deanei CBS134746 T
 Readeriella menaiensis CBS125003 T
 Meristemomyces frigidus CCFEE 5508
 Apenidiella strumelloidea CBS114484
 Oleoguttula mirabilis CCFEE 5522 T
 Phaeothecoidea minutispora CPC13710 T
 Phaeothecoidea intermedia CBS124994 T
 Neotrimmatostroma excentricum CBS121102 T
 Suberoteratosphaeria xenosuberosa CBS134747 T
 Acrodontium crateriforme CPC 11509
 Acrodontium pigmentosum CBS 111111 T
 Neodevriesia agapanthi CBS 132689 T
 Neodevriesia strelitziae CBS 122379 T
 Piedraia hortae CBS480.64
 Parapenidiella tasmaniensis CBS111687 T
 Teratoramularia infinita CPC 19488 T
-/-/0.97
85/-/0.98
98/-/0.99
-/-/1
 Teratoramularia rumicicola CPC 14653 T
 Myrtapenidiella corymbia CBS124769 T
 Incertomyces perditus CCFEE 5385 T
 Staninwardia suttonii CBS120061 T
0.10
