# Supplementary material for: Fungal Diversity Associated with Thirty-Eight Lichen Species Revealed a New Genus of Endolichenic Fungi, Intumescentia gen. nov. (Teratosphaeriaceae)
Source: J Fungi (Basel). 2023 Mar 29;9(4):423. doi: 10.3390/jof9040423 (PMC10143819; doi:10.3390/jof9040423)
Supplement: Supplementary file 1 [file jof-09-00423-s001.zip › Figure S6 ACT.pptx]

## Slide 1
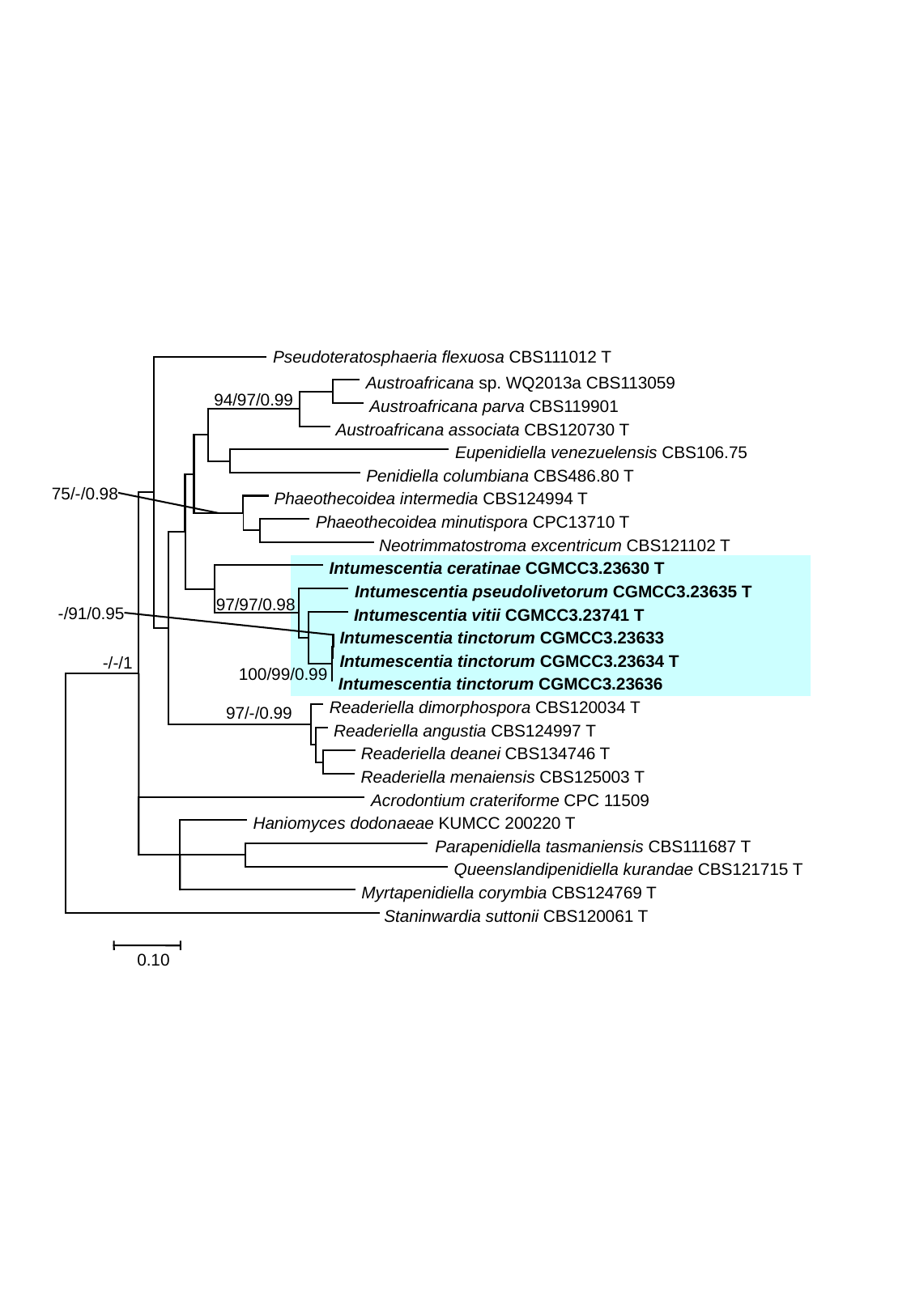

Pseudoteratosphaeria flexuosa CBS111012 T
 Austroafricana sp. WQ2013a CBS113059
94/97/0.99
 Austroafricana parva CBS119901
 Austroafricana associata CBS120730 T
 Eupenidiella venezuelensis CBS106.75
 Penidiella columbiana CBS486.80 T
75/-/0.98
 Phaeothecoidea intermedia CBS124994 T
 Phaeothecoidea minutispora CPC13710 T
 Neotrimmatostroma excentricum CBS121102 T
 Intumescentia ceratinae CGMCC3.23630 T
 Intumescentia pseudolivetorum CGMCC3.23635 T
97/97/0.98
-/91/0.95
 Intumescentia vitii CGMCC3.23741 T
 Intumescentia tinctorum CGMCC3.23633
 Intumescentia tinctorum CGMCC3.23634 T
-/-/1
100/99/0.99
 Intumescentia tinctorum CGMCC3.23636
 Readeriella dimorphospora CBS120034 T
97/-/0.99
 Readeriella angustia CBS124997 T
 Readeriella deanei CBS134746 T
 Readeriella menaiensis CBS125003 T
 Acrodontium crateriforme CPC 11509
 Haniomyces dodonaeae KUMCC 200220 T
 Parapenidiella tasmaniensis CBS111687 T
 Queenslandipenidiella kurandae CBS121715 T
 Myrtapenidiella corymbia CBS124769 T
 Staninwardia suttonii CBS120061 T
0.10
