# Supplementary material for: Fungal Diversity Associated with Thirty-Eight Lichen Species Revealed a New Genus of Endolichenic Fungi, Intumescentia gen. nov. (Teratosphaeriaceae)
Source: J Fungi (Basel). 2023 Mar 29;9(4):423. doi: 10.3390/jof9040423 (PMC10143819; doi:10.3390/jof9040423)
Supplement: Supplementary file 1 [file jof-09-00423-s001.zip › Table S2.docx]

**Table S2** Taxa used in the phylogenetic analyses and their corresponding GenBank numbers.

| **Species** | **Strain** | **GenBank Accession Numbers** | | | | | | |
| --- | --- | --- | --- | --- | --- | --- | --- | --- |
|  |  | **ITS** | **LSU** | **SSU** | **ACT** | **CAL** | **RPB2** | **TEF1** |
| *Acidiella americana* | CCF 5435 ^T^ | LT627242 | LT627241 | - | - | - | - | LR778331 |
| *Acidiella bohemica* | CBS 132720 ^T^ | - | KF901984 | - | - | - | KF902178 | - |
| *Acidiella polonica* | CCF 6237^T^ | MK271751 | MN319582 | - | - | - | LR778336 | LR778326 |
| *Acidiella uranophila* | CBS 136962^T^ | JQ904602.2 | KF857170 | - | - | - | - | - |
| *Acidomyces acidophilus* | CBS 272.74 | MH860851 | MH872589 | - | - | - | - | - |
| *Acrodontium crateriforme* | CPC 11509 | GU214682 | GU214682 | - | GU320413 | KX289011 | KX288404 | GU384425 |
| *Acrodontium pigmentosum* | CBS 111111 ^T^ | KX287275 | KX286963 | - | - | - | KX288412 | - |
| *Apenidiella strumelloidea* | CBS 114484 | - | KF937229 | - | - | - | KF937266 | - |
| *Araucasphaeria foliorum* | CPC 33084 ^T^ | MH327793 | MH327829 | - | - | - | - | - |
| *Austroafricana associata* | CPC 13119 ^T^ | KF901507 | KF901824 | - | KF903526 | KF902528 | KF902177 | KF903087 |
| *Austroafricana parva* | CMW 10189 | - | KF251778 | - | KF903512 | KF902537 | KF902192 | KF903097 |
| *Austroafricana sp.* | CPC 4313 | KF901498 | KF901813 | - | KF903460 | KF902527 | KF902186 | KF903086 |
| *Austrostigmidium mastodiae* | MA 18215 ^T^ | - | NG057063 | - | - | - | - | - |
| *Batcheloromyces alistairii* | CPC 12730 ^T^ | - | KF937220 | - | - | - | KF937252 | - |
| *Batcheloromyces leucadendri* | CPC 1838 ^T^ | - | KF937221 | - | - | - | KF937253 | - |
| *Batcheloromyces sedgefieldii* | CPC 3026 ^T^ | - | KF937222 | - | - | - | KF937254 | - |
| *Baudoinia antiliensis* | UAMH 10810 | NR153616 | NG058686 | - | - | - | - | KT186546 |
| *Baudoinia caledoniensis* | UAMH 10761 ^T^ | NR153614 | NG058685 | - | - | - | - | KT186538 |
| *Baudoinia compniacensis* | UAMH 10808 | NR153612 | KT186489 | - | - | - | - | KT186533 |
| *Bryochiton monascus* | CBS 126284 | MH863957 | MH875416 | - | - | - | - | - |
| *Caatingomyces brasilensis* | URM 7916 | MH929437 | MH929439 | - | - | - | MH929441 | MH929443 |
| *Camarosporula persooniae* | CPC 3350 | - | JF770460 | - | - | - | KF937255 | - |
| *Capnobotryella renispora* | CBS 21490 ^T^ | NR121295 | GU214399 | - | - | - | - | - |
| *Catenulostroma hermanusense* | CPC 18276 ^T^ | - | KF902089 | - | - | - | KF902197 | - |
| *Catenulostroma protearum* | CPC 15370 ^T^ | - | KF902090 | - | - | - | KF902198 | - |
| *Constantinomyces minimus* | CBS 118766 ^T^ | NR144957 | KF310003 | - | - | - | KF310077 | - |
| *Elasticomyces elasticus* | CCFEE 5313 ^T^ | FJ415474 | KJ380894 | - | - | - | - | - |
| *Eupenidiella venezuelensis* | CBS 106.75 | KF901802 | KF902163 | - | KF903393 | KF902540 | KF902202 | KF903100 |
| *Euteratosphaeria verrucosiafricana* | CPC 11167 ^T^ | DQ303056 | - | - | - | - | - | - |
| *Friedmanniomyces endolithicus* | CCFEE 5283 | - | KF310006 | - | - | - | KF310053 | - |
| *Haniomyces dodonaeae* | KUMCC20-0220 ^T^ | MW264212 | MW264191 | - | MW256802 | MW256805 | MW269527 | MW256813 |
| *Hispidoconidioma alpina* | UAMH 11010 | FJ997285 | FJ997286 | - | - | - | - | - |
| *Hortaea thailandica* | CPC 16651 | - | KF902125 | - | - | - | KF902206 | - |
| *Hyweljonesia indica* | NFCCI 4146 ^T^ | NR164021 | NG066398 | - | - | - | - | - |
| *Hyweljonesia queenslandica* | BRIP 61322b ^T^ | NR154095 | NG059766 | - | - | - | - | - |
| *Incertomyces perditus* | CCFEE 5385 | KF309977 | KF310008 | - | - | - | KF310083 | - |
| *Incertomyces vagans* | CCFEE 5393 ^T^ | NR154064 | KF310009 | - | - | - | KF310057 | - |
| ***Intumescentia ceratinae*** | **CGMCC3.23630 ^T^** | **OP342838** | **OP345117** | **OP345112** | **OP354480** | **OP354486** | **OP354474** | **OP354468** |
| ***Intumescentia pseudolivetorum*** | **CGMCC3.23635** **^T^** | **OP345109** | **OP345119** | **OP345148** | **OP354479** | **OP354485** | **OP354473** | **OP354467** |
| ***Intumescentia tinctorum*** | **CGMCC3.23634** **^T^** | **OP345149** | **OP345116** | **OP345114** | **OP354478** | **OP354484** | **OP354472** | **OP354466** |
| ***Intumescentia tinctorum*** | **CGMCC3.23633** | **OP345115** | **OP345111** | **OP345113** | **OP354477** | **OP354483** | **OP354471** | **OP354465** |
| ***Intumescentia tinctorum*** | **CGMCC3.23636** | **OP289531** | **OP326178** | **OP326174** | **OP354476** | **OP354482** | **OP354470** | **OP354464** |
| ***Intumescentia vitii*** | **CGMCC3.23741** **^T^** | **OP342841** | **OP345120** | **OP345110** | **OP354481** | **OP354487** | **OP354475** | **OP354469** |
| *Lapidomyces hispanicus* | TRN126 | - | KF310016 | - | - | - | KF310076 | - |
| *Meristemomyces frigidum* | CCFEE 5457 | - | GU250389 | - | - | - | KF310066 | - |
| *Microcyclospora pomicola* | CBS 126141 ^T^ | NR160241 | NG064231 | - | - | - | - | - |
| *Monticola elongata* | CCFEE 5492 | - | KF309994 | - | - | - | KF310065 | - |
| *Myrtapenidiella corymbia* | CPC 14640 ^T^ | KF901517 | KF901838 | - | KF903558 | KF902558 | KF902227 | KF903119 |
| *Neocatenulostroma abietis* | CBS 110038 | - | KF937226 | - | - | - | KF937263 | - |
| *Neocatenulostroma microsporum* | CPC 1960 ^T^ | KF901499 | KF901814 | - | - | KF902561 | KF902232 | KF903122 |
| *Neodevriesia agapanthi* | CPC 19833 ^T^ | - | JX069859 | - | - | - | KJ564346 | - |
| *Neodevriesia strelitziae* | X1037 ^T^ | EU436763 | GU301810 | - | - | - | GU371738 | GU349049 |
| *Neophaeothecoidea proteae* | CPC 2831 ^T^ | - | KF937228 | - | - | - | KF937265 | - |
| *Neotrimmatostroma excentricum* | CPC 13092 ^T^ | KF901518 | KF901840 | - | KF903534 | KF902562 | KF902236 | KF903123 |
| *Oleoguttula mirabilis* | CCFEE 5522 ^T^ | - | KF310019 | - | - | - | KF310070 | - |
| *Parapenidiella tasmaniensis* | CPC 1555 ^T^ | KF901521 | KF901843 | - | KF903451 | KF902587 | KF902263 | KF903150 |
| *Parateratosphaeria persooni* | CBS 122895 ^T^ | NR145096 | NG058074 | - | - | - | - | - |
| *Parateratosphaeria stirlingloe* | CPC 29252 ^T^ | NR155699 | NG059800 | - | - | - | - | KY979890 |
| *Parateratosphaeria marasasi* | CBS 122899 ^T^ | NR145095 | NG058073 | - | - | - | KF937268 | - |
| *Penidiella aggregata* | CBS 128772 ^T^ | NR137772 | NG057905 | - | - | - | - | - |
| *Penidiella columbiana* | CBS 486.80 ^T^ | NR156579 | NG057774 | - | KF903587 | KF902594 | KF902272 | KF903158 |
| *Penidiella drakensbergensis* | CPC 19778 ^T^ | NR111821 | NG059482 | - | - | - | - | - |
| *Penidiellopsis radicularis* | CBS 131976 | KT833148 | KU216314 | - | - | KU216292 | - | KU216339 |
| *Penidiellopsis ramosus* | CBMAI 1937 | KT833151 | KU216317 | - | - | KU216295 | - | KU216342 |
| *Phaeothecoidea intermedia* | CPC 13711 ^T^ | KF901752 | KF902106 | - | KF903564 | KF902606 | KF902286 | KF903171 |
| *Phaeothecoidea minutispora* | CPC 13710 ^T^ | KF901753 | KF902108 | - | KF903659 | KF902607 | KF902288 | KF903172 |
| *Piedraia hortae var. hortae* | CBS 480.64 | - | KF901943 | - | - | - | KF902289 | - |
| *Piedraia hortae var. paraguayensis* | CBS 276.32 | - | KF901816 | - | - | - | - | - |
| *Piedraia quintanilhae* | CBS 327.63 ^T^ | - | KF901957 | - | - | - | - | - |
| *Pseudotaenlolina globosa* | CBS 10989 ^T^ | NR136960 | NG057777 | - | - | - | MW371115 | - |
| *Pseudoteratosphaeria flexuosa* | CPC 1109 ^T^ | KF901755 | KF902110 | - | KF903421 | KF902654 | KF902346 | - |
| *Queenslandipenidiella kurandae* | CPC 13333 ^T^ | KF901538 | KF901860 | - | KF903538 | KF902663 | KF902356 | KF903238 |
| *Readeriella angustia* | CPC 13608 ^T^ | KF901759 | KF902114 | - | KF903566 | KF902669 | KF902364 | KF903246 |
| *Readeriella deanei* | CPC 12715 ^T^ | KF901542 | KF901864 | - | KF903583 | KF902673 | KF902368 | KF903250 |
| *Readeriella dimorphospora* | CPC 12636 ^T^ | KF901544 | KF901866 | - | KF903622 | KF902675 | KF902370 | KF903252 |
| *Readeriella menaiensis* | CPC 14447 ^T^ | KF901548 | KF901870 | - | KF903572 | KF902678 | KF902374 | KF903256 |
| *Recurvomyces mirabilis* | CCFEE 5264 ^T^ | - | GU250372 | - | - | - | KF310059 | - |
| *Salinomyces thailandica* | CBS125423 ^T^ | NR171710 | NG057846 | - | - | - | KF902206 | - |
| *Staninwardia suttonii* | CBS120061 | NR171710 | NG057846 | - | - | - | KF902206 | - |
| *Simplicidiella nigra* | CBMAI 1939 | KT833147 | KU216313 | - | - | KU216291 | - | KU216338 |
| *Staninwardia suttonii* | CPC 13055 ^T^ | KF901552 | KF901874 | - | KF903517 | KF902693 | KF902392 | KF903270 |
| *Stenella araguata* | FMC 245 | - | KF902168 | - | - | - | KF902393 | - |
| *Suberoteratosphaeria pseudosuberosa* | CPC 12085 ^T^ | KF901786 | KF902144 | - | KF903508 | - | - | KF903275 |
